# RedB, a Member of the CRP/FNR Family, Functions as a Transcriptional Redox Brake

**DOI:** 10.1128/spectrum.02353-22

**Published:** 2022-09-15

**Authors:** Nijia Ke, Joseph E. Kumka, Mingxu Fang, Brian Weaver, Judith N. Burstyn, Carl E. Bauer

**Affiliations:** a Molecular and Cellular Biochemistry Department, Indiana University, Bloomington, Indiana, USA; b Department of Chemistry, University of Wisconsin—Madison, Madison, Wisconsin, USA; Griffith University

**Keywords:** CRP/FNR ortholog, transcriptomics, global regulation, limiting energy production

## Abstract

Phylogenetic and sequence similarity network analyses of the CRP (cyclic AMP receptor protein)/FNR (fumarate and nitrate reductase regulatory protein) family of transcription factors indicate the presence of numerous subgroups, many of which have not been analyzed. Five homologs of the CRP/FNR family are present in the Rhodobacter capsulatus genome. One is a member of a broadly disseminated, previously uncharacterized CRP/FNR family subgroup encoded by the gene *rcc01561*. In this study, we utilize mutational disruption, transcriptome sequencing (RNA-seq), and chromatin immunoprecipitation sequencing (ChIP-seq) to determine the role of RCC01561 in regulating R. capsulatus physiology. This analysis shows that a mutant strain disrupted for *rcc01561* exhibits altered expression of 451 genes anaerobically. A detailed analysis of the affected loci shows that RCC01561 represses photosynthesis and favors catabolism over anabolism and the use of the Entner-Doudoroff shunt and glycolysis over that of the tricarboxylic acid (TCA) cycle to limit NADH and ATP formation. This newly characterized CRP/FNR family member with a predominant role in reducing the production of reducing potential and ATP is given the nomenclature RedB as it functions as an energy and redox brake. Beyond limiting energy production, RedB also represses the expression of numerous genes involved in protein synthesis, including those involved in translation initiation, tRNA synthesis and charging, and amino acid biosynthesis.

**IMPORTANCE** CRP and FNR are well-characterized members of the CRP/FNR family of regulatory proteins that function to maximize cellular energy production. In this study, we identify several new subgroups of the CRP/FNR family, many of which have not yet been characterized. Using Rhodobacter capsulatus as a model, we have mutationally disrupted the gene *rcc01561*, which codes for a transcription factor that is a member of a unique subgroup of the CRP/FNR family. Transcriptomic analysis shows that the disruption of *rcc01561* leads to the altered expression of 451 genes anaerobically. Analysis of these regulated genes indicates that RCC01561 has a novel role in limiting cellular energy production. To our knowledge, this is first example of a member of the CRP/FNR family that functions as a brake on cellular energy production.

## INTRODUCTION

The CRP (cyclic AMP receptor protein)/FNR (fumarate and nitrate reductase regulatory protein) regulatory family constitutes a large collection of transcription factors that are widespread among diverse groups of bacteria (1, 2). This family of transcription factors shares similar structural features comprised of an N-terminal cofactor binding domain followed by a dimerization connection helix and then a C-terminal helix-turn-helix (HTH) DNA binding motif (3, 4). One of the better-characterized members of this family is CRP, which senses changes in cAMP concentrations via the formation of a cAMP-CRP cocomplex. The binding of cAMP promotes stable dimer formation and the movement of a helix-turn-helix domain into a position that promotes binding to target DNA sequences (5, 6). The cAMP-CRP complex regulates over 100 genes involved in energy metabolism in response to the presence of cAMP (7). Another well-characterized member is FNR, which contains an oxygen-labile 4-iron–4-sulfur cluster (8). Under anaerobic conditions, the 4Fe-4S complex is stable, with its formation promoting dimerization and subsequent DNA binding (9, 10). Under aerobic conditions, the oxidation of the 4Fe-4S complex leads to its disassembly, causing disruptions of FNR dimerization and subsequent DNA binding activity (11). Like CRP, FNR is a global regulator of many genes involved in energy production, particularly those required for anaerobic growth, such as fumarate reductase and nitrite and nitrate reductase, etc. (12). The other well-characterized member is the carbon monoxide-sensing regulator (CooA), which utilizes heme as a cofactor for binding CO and affects dimerization and DNA binding activity (13, 14). CooA is not a global regulator but instead regulates an operon coding for carbon monoxide dehydrogenase, which oxidizes CO to CO_2_ (15). These three examples highlight the fact that members of this family have evolved diverse mechanisms of sensing effectors that control their activity and their diverse roles in controlling cellular physiology. However, there are still many phylogenetically distinct subgroups in this family that are as yet uncharacterized.

This study focuses on our continued analysis of the control of anaerobic metabolism by the purple nonsulfur photosynthetic bacterium Rhodobacter capsulatus. This species is metabolically versatile, allowing growth under a wide variety of conditions. For example, these cells can grow heterotrophically under dark aerobic conditions using organic carbon sources for respiratory energy production. Under dark conditions, they can grow via anaerobic respiration using carbon sources coupled with either dimethyl sulfoxide (DMSO) or nitrite as the terminal electron acceptor (16). Under anaerobic light conditions, these cells can grow either photoheterotrophically with an organic carbon source or photoautotrophically using the Calvin-Benson-Bassham (CBB) pathway to fix CO_2_ into cellular carbon (17). Coordinating various metabolic shifts that promote optimal growth under different environmental conditions must rely heavily on signal transduction pathways and transcription factors that can sense and appropriately respond to environmental changes. This supposition is supported by the observation that ~53% of the R. capsulatus genome undergoes changes in gene expression when cells shift from aerobic to anaerobic growth conditions (18).

The R. capsulatus genome codes for five members of the CRP/FNR family (FnrL, RCC01561, RCC01361, RCC00574, and RCC03255). In this species, a member of the FNR subgroup, called FnrL, is the only one that has been extensively characterized (19). Transcriptomic analyses indicate that like FNR from Escherichia coli, FnrL is also a global regulator that directly and indirectly controls the expression of ~807 genes, mainly via the anaerobic activation of gene expression (19). In this study, we have undertaken genetic and transcriptome analyses of a novel member of a CRP/FNR subgroup (*rcc01561*) that, to our knowledge, has not been previously analyzed. The mutational disruption of *rcc01561* leads to the altered expression of 451 genes anaerobically. Analysis of the affected loci indicates that RCC01561 represses the expression of the photosystem and alters central metabolism in a manner that limits energy and protein production. Consequently, we have named this newly characterized member of this novel subgroup of the CRP/FNR family RedB, for “redox brake.”

## RESULTS AND DISCUSSION

### RedB is a unique, uncharacterized member of the CRP/FNR superfamily.

**FIG 1:**
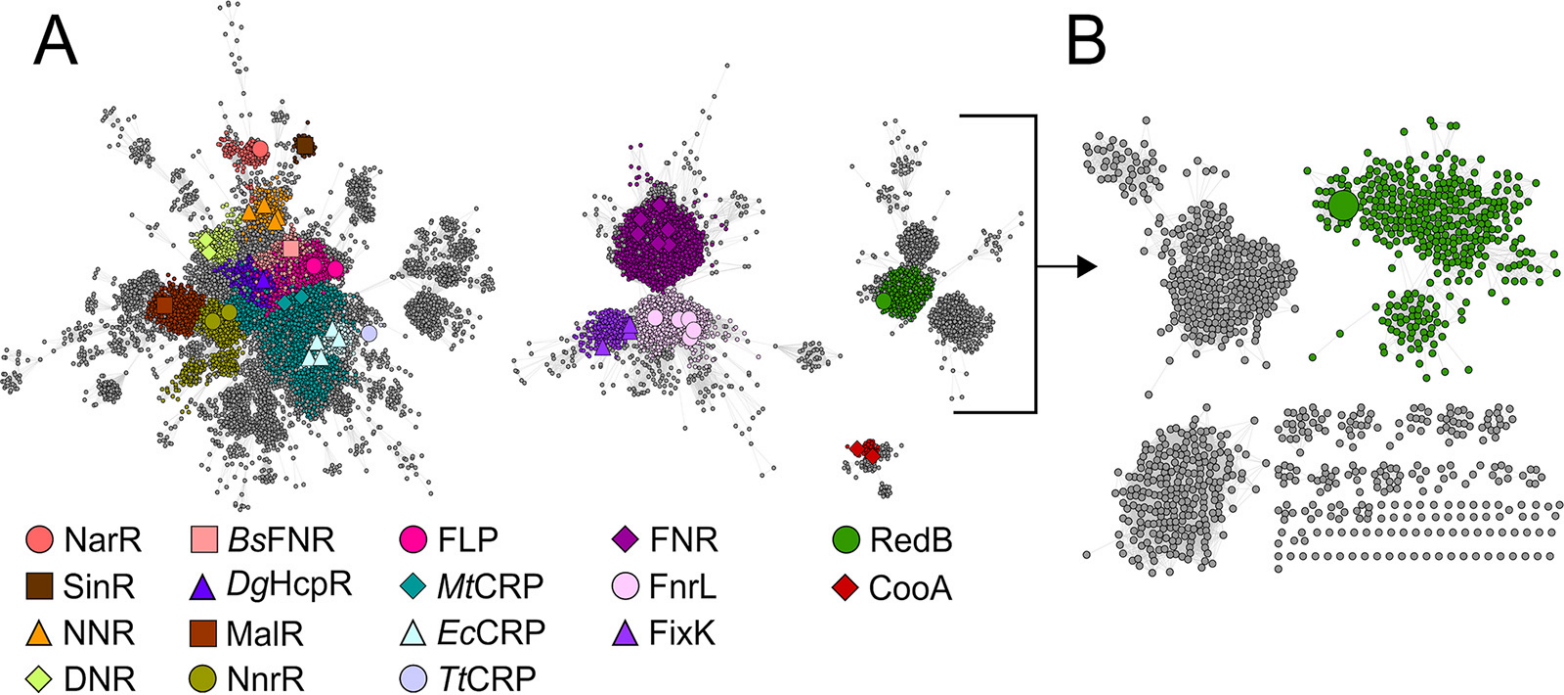
Sequence similarity network of the CRP/FNR superfamily depicting the most well-characterized proteins. (A) Partial CRP/FNR superfamily sequence similarity network depicting four selected clusters at a cutoff score of 40 (38% identity). Subclusters containing a characterized CRP/FNR transcription factor are colored according to the key at the bottom. *Ec*CRP, E. coli CRP. (B) RedB subcluster from panel A increased to an alignment cutoff score of 49 (43% identity). The complete sequence similarity network for the CRP/FNR superfamily is shown in Fig. S1 and S2 in the supplemental material. UniProt accession numbers for characterized CRP/FNR proteins are included in Table S1. EcCRP, *Escherichia coli* cyclic AMP receptor protein; NNR, nitrite reductase and nitric oxide reductase regulator; DNR, dissimilative nitrate respiration regulator; *Bs*FNR, *Bacillus subtilis* fumarate and nitrate reduction regulator; *Dg*HcpR, *Desulfovibrio gigas* hybrid cluster protein regulator; *Mt*CRP, *Mycobacterium tuberculosis* cyclic AMP receptor protein; *Tt*CRP, *Thermus thermophilus* cyclic AMP receptor protein.

To better understand RedB and its relationship with other CRP/FNR proteins, we constructed a sequence similarity network (SSN). We curated a list of 97,535 CRP/FNR proteins from the InterPro database (20) and generated a sequence similarity network by inputting these sequences into the Enzyme Function Initiative-Enzyme Similarity Tool (EFI-EST) (21). We mapped previously characterized CRP/FNR proteins (see Table S1 in the supplemental material) onto the network and increased the alignment cutoff stringency until these characterized proteins were separated into isofunctional clusters. Even at a low cutoff score of 40 (Fig. 1 and Fig. S1 and S2), there are many clusters present in this network, indicating that the CRP/FNR superfamily is highly diverse. This diversity of the CRP/FNR superfamily has been previously demonstrated by phylogenetic analyses (4) and was expected given the significant variety of effector molecules sensed by this diverse family of transcription factors. Upon mapping RedB onto our network, our sequence similarity network analysis reveals that RedB does not cluster with any previously characterized CRP/FNR protein at any cutoff score of 40 or higher (Fig. 1 and Fig. S1 and S2). This observation indicates that RedB represents a new subgroup of CRP/FNR transcription factors with heretofore-unknown biological function and regulatory activity.

### Identifying genes that are directly and indirectly regulated by RedB.

We identified genes regulated by RedB by performing transcriptome sequencing (RNA-seq) analysis where we compared the gene expression levels in the Δ*redB* strain to the expression levels in wild-type R. capsulatus. Three biological replicates of each strain were grown under anaerobic (photosynthetic) conditions to the early/mid-log growth phase, from which RNA was extracted for RNA-seq analysis as described in Materials and Methods.

As previously reported for the FnrL regulon (19), the regulatory effects of RedB were also observed to be relatively weak. Thus, genes were called as being differentially expressed genes (DEGs) in the Δ*redB* strain, relative to wild-type cells, when they exhibited a log_2_ fold change of no less than +0.25 or no more than −0.4, with an adjusted *P* value of less than 0.05. Finally, genes with average normalized counts of <100 were excluded to ensure that called genes exhibited a basal level of expression. Using these criteria, RedB was found to regulate 451 genes under anaerobic conditions (Tables S2 and S3). Among these, 244 were activated by RedB, and 207 were repressed by RedB.

**FIG 2:**
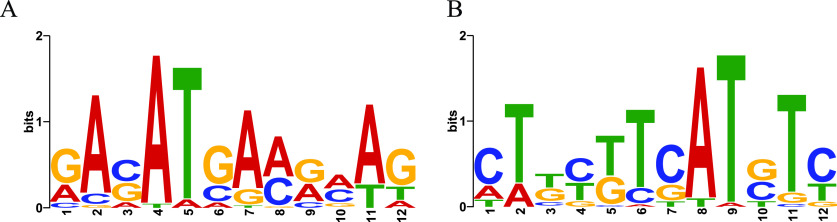
DNA binding motifs of RedB orthologs. The consensus motifs were determined using the MEME tool based on the sequences of ChIP-seq-called peaks. (A) The consensus motif in the forward direction. (B) The consensus motif in the reverse complementary direction.

We also undertook chromatin immunoprecipitation sequencing (ChIP-seq) analysis to further differentiate genes that are directly regulated by RedB from those that are indirectly regulated. ChIP-seq peaks were called if they were present in three biological replicates exhibiting a false discovery rate of <5%. With this as a criterion, a total of 115 significant peaks were called as representing putative RedB binding sites on the genome (Tables S2 and S4). A consensus DNA recognition motif for RedB was obtained using MEME to search for related sequences in these called ChIP-seq peaks. As shown in Fig. 2, the putative recognition sequence identified by MEME is a nonpalindromic sequence of 12 bp in which the 4th, 5th, and 11th bases are comprised of A or T, with A being present in 96.7%, 6.7%, and 76.6% of the called binding sites, respectively. At both the 2nd and 7th positions, an A base is also dominant, with 86.7% and 80% likelihoods, respectively, with the three other bases occupying these positions at lower frequencies. It is notable that R. capsulatus has a very high GC content (66.6%) (22), so the presence of such a highly AT-rich consensus motif in the called ChIP-seq peaks provides further support for this putative MEME-derived consensus sequence. Furthermore, asymmetrical (nonpalindromic) DNA binding recognition sequences have been observed with another member of the CRP family that also contains AT-rich regions (23). It has been proposed that an asymmetric AT-rich site promotes a flexible “bend” in the DNA that allows the DNA strands to contact regions beyond that of the helix-turn-helix domain, resulting in a nonpalindromic recognition sequence (23).

**FIG 3:**
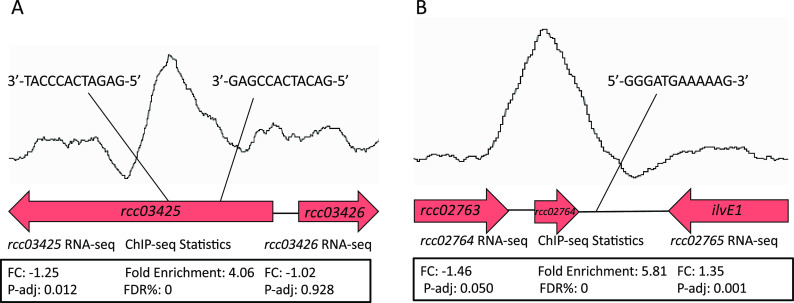
Representative RedB ChIP-seq peaks with nearby genes undergoing alterations in expression upon the deletion of RedB. (A) ChIP-seq peak located within the coding region of *rcc03425* that is repressed by RegB, as indicated by RNA-seq. (B) ChIP-seq peak located between the coding regions of *rcc02764* and *ilvE1*, with RNA-seq analysis indicating that RegB represses the expression of *ilvE1*. FC, fold change; P-adj, adjusted *P* value; FDR, false discovery rate.

Comparative analysis of the ChIP-seq and RNA-seq data sets shows that among the 115 called ChIP-seq peaks, 47 peaks (41%) were located upstream of, downstream of, or within a gene or genes that exhibited differential expression based on RNA-seq analysis. Further analysis of these peak locations shows that the expression of 63 genes appears to be directly regulated by RedB, with 24 being directly activated and 39 being directly repressed (Table S2). Fig. 3 shows two examples of ChIP-seq peaks with nearby genes being regulated by RedB. In Fig. 3A, a RedB ChIP-seq peak is located in the middle of *rcc03425*, a gene whose expression is upregulated by RedB by 1.25-fold. In this peak, there are two putative RedB recognition sequences, as indicated. In Fig. 3B, we show a RedB ChIP-seq peak within *rcc02764* that is located between the coding regions of both *rcc02764* and *ilvE1* (69 bp downstream of *rcc02764* and 298 bp downstream of *ilvE1*). At this location, there is a single putative RedB recognition sequence. RedB thus appears to directly repress the expression of *ilvE1*, which codes for an aminotransferase, by 1.35-fold.

### COG assignment of RedB regulons.

**FIG 4:**
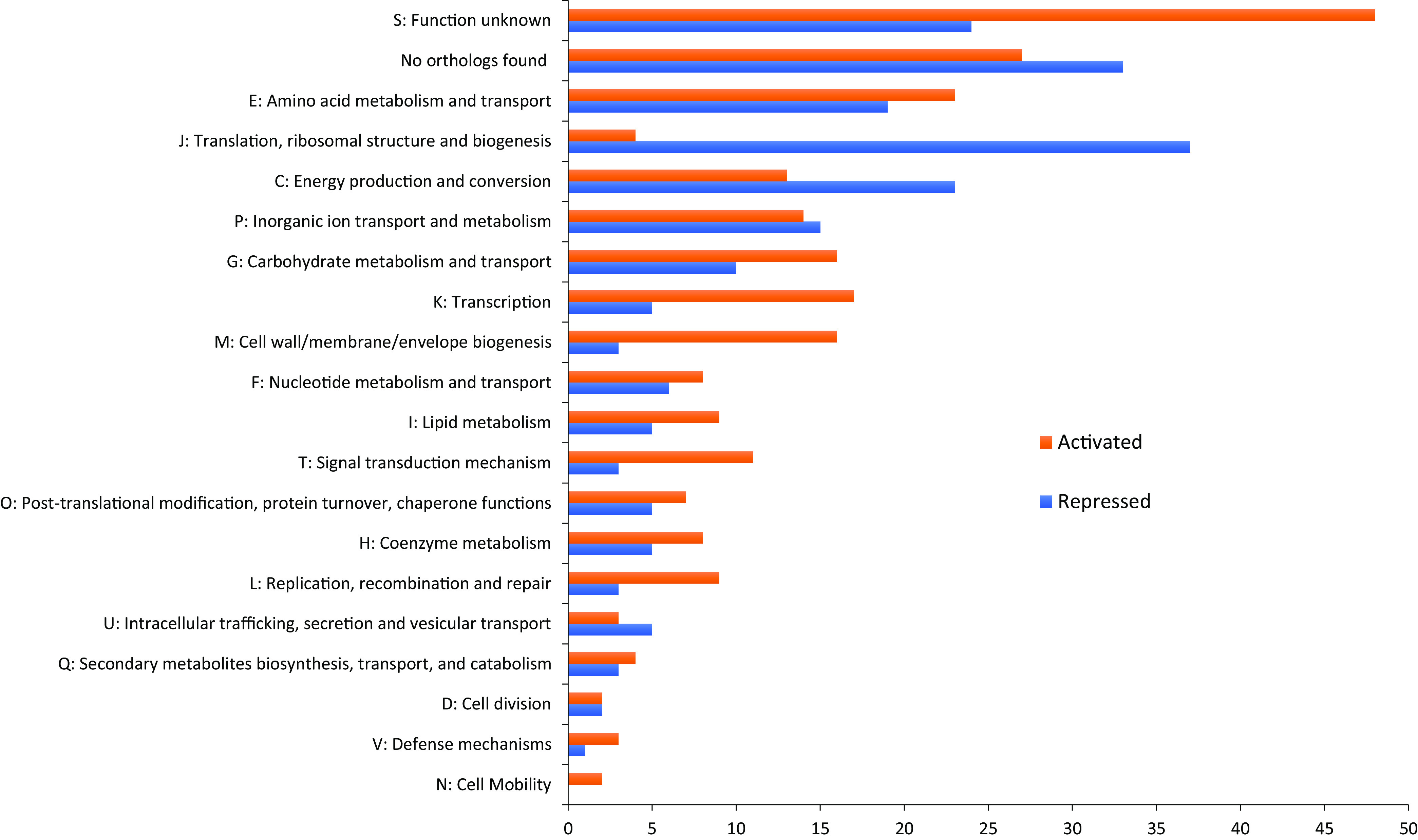
Histogram showing the number of RedB regulons classified into each COG. The bars are ordered based on descending order of the total number of genes in each COG. Red denotes RedB-activated genes exhibiting decreases in expression levels in the Δ*redB* strain, and blue represents RedB-repressed genes that show increased expression levels in the Δ*redB* strain.

We investigated which pathways or cellular activities are regulated by RedB by classifying genes into Clusters of Orthologous Groups (COGs) categories. As shown in the bar graph in Fig. 4, genes encoding proteins of unknown function (COG S) or those having no corresponding orthologs are categories that have the highest numbers of genes (15.5% and 14.0% of the RedB regulon, respectively). Apart from these two categories, COG E shows that RedB has an active role in regulating amino acid metabolism and transport, with a slightly lower number of upregulated than downregulated genes. Another notable COG is COG J, which contains a large number of genes involved in translation, ribosomal structure, and biogenesis. Interestingly, RedB repressed the expression of most genes in this COG (34 genes), while it activated only 4 genes. The other COGs that attract attention are COGs K and T, which contain large numbers of transcription (22 genes, or 4.9%) and signal transduction (14 genes, or 3.1%) genes. In both regulatory COGs, there are many more genes in this group that exhibit decreased expression than those that exhibit upregulated expression upon the disruption of RedB. This indicates that RedB has a broad role in activating the expression of numerous regulatory proteins. Below, we discuss in detail the role of RedB in either the direct or indirect control of notable cellular processes.

### RedB directly regulates several transcription factors and signal transduction components.

As shown in the COG K subgroup in Fig. 4 and in detail in Tables S2 and S3, RedB regulates the expression of 22 transcription factors according to ChIP-seq analysis, indicating that three members of this group are directly regulated by RedB (Table S2). Specifically, RedB activates the synthesis of a LysR family transcriptional regulator and a helix-turn-helix domain-containing protein encoded by *rcc02527*. RedB also directly represses the synthesis of a CarD family transcriptional regulator by binding at the beginning of *carD* from bp +6 to bp +17 as predicted by the FIMO tool. CarD is a global regulator that activates the expression of a number of genes, especially those involved in rRNA synthesis (24). This is consistent with our finding (discussed below) that RedB indirectly downregulates a plethora of genes involved in translation.

In addition to transcription regulators, RedB also directly and indirectly regulates 14 genes in the COG T signal transduction subgroup (Fig. 4 and Tables S2 and S3). Of note, RedB indirectly activates the expression of two diguanylate cyclases/phosphodiesterases (*rcc01110* and *rcc00783*), a histidine kinase (*hupT*) involved in hydrogen uptake (25), and a response regulator involved in the expression of genes for cytochrome *bc*_1_ (*petR*) (26). RedB also indirectly represses the expression of a response regulator involved in regulating phosphate uptake (*phoB*) (Table S3). Regarding direct control (Table S2), RedB directly activates the expression of *dorS*, which encodes the DMSO-TMAO sensor hybrid histidine kinase (27), as there are two ChIP-seq peaks upstream of *dorS*. In the presence of trimethylamine N-oxide (TMAO), DorS phosphorylates the transcriptional regulator DorR, which then upregulates the expression of *torA*, which codes for the terminal electron acceptor TMAO reductase (28, 29). Interestingly, the RNA-seq results also show that RedB downregulates the expression of *torA* 1.33-fold instead of upregulating it. Thus, other transcription factors regulated by RedB likely inactivate the expression of *torA*, which may override the activation effects of *torA* by DorR.

An additional regulatory gene that RedB directly activates is *glnE*, which encodes a bifunctional enzyme that regulates the activity of the glutamine synthetase GlnA through adenylylation/deadenylylation (Table S2) (30). When the nitrogen level is high in the environment, GlnE inactivates GlnA by transferring an adenylyl group from ATP to GlnA. When the nitrogen level is low, GlnE deadenylylates GlnA, which activates ammonia assimilation activity (31). Furthermore, in addition to directly activating the expression of *glnE*, RedB also indirectly activates *glnA1* expression (Table S3). This potentially assists in magnifying the regulatory effect of GlnE. Overall, it appears that RedB is both directly and indirectly involved in maintaining nitrogen equilibrium inside these cells.

### RedB limits photosynthesis energy production.

Our RNA-seq and ChIP-seq results indicate that RedB directly represses the expression of *pucDE*, which encode subunits of the light-harvesting B-800/850 complex (32) (Table S2). RedB also indirectly represses the expression of both *pufA*, encoding the alpha subunit of the light-harvesting protein B-870, and *puhA*, coding for subunit H of the photosynthetic reaction center (Table S3) (33). Therefore, it can be inferred that RedB inhibits the synthesis of the R. capsulatus photosystem.

**FIG 5:**
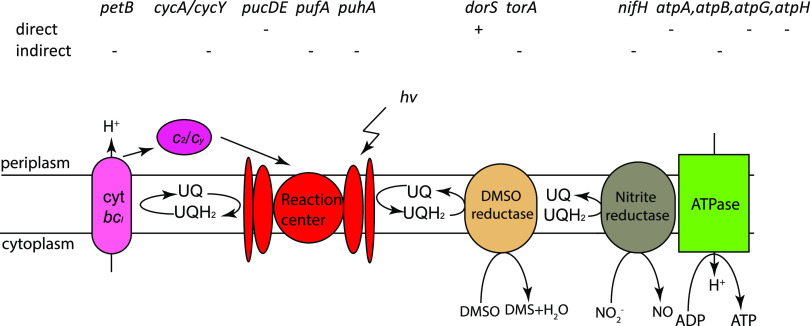
Schematic of R. capsulatus anaerobic cellular activities taking place on the intracytoplasmic membrane that are regulated by RedB. The activation or repression of genes is indicated by + or −, respectively. Genes directly or indirectly regulated by RedB are indicated separately. cyt, cytochrome; UQ, ubiquinone; UQH_2_, reduced ubiquinol hv, solar energy; DMSO, dimethyl sulfoxide; DMS, dimethyl sulfide.

Protons are pumped out of the inner membrane during photosynthesis and respiration when electrons pass through the *bc*_1_ complex of the electron transport chain (34). The resultant proton gradient is subsequently used to generate ATP via ATPase (Fig. 5). As discussed above, RedB affects the expression of peptide components of the photosystem, but it indirectly activates the expression of the response regulator PetR, which is need for the expression of the *pet* operon coding for peptide components of the *bc*_1_ complex (Table S3) (26). In addition, RedB directly downregulates the expression of *hemA*. encoding 5-aminolevulinate (ALA) synthase, which converts glycine into ALA, the initial and rate-limiting step in the tetrapyrrole biosynthetic pathway (Table S2) (35). This pathway leads to the biosynthesis of heme, which is the essential electron carrier cofactor in cytochromes, and also the biosynthesis of bacteriochlorophyll *a*, which is the essential electron-donating component of the photosystem (36).

Another critical component for electron transport in photosynthesis, and respiration, is the electron and proton carrier ubiquinone. Ubiquinone is reduced and protonated by the light-excited photosystem and subsequently passes electrons and protons to cytochrome *bc*_1_ (Fig. 5). It is therefore notable that RedB indirectly inhibits ubiquinol biosynthesis by repressing the expression of *ubiG*, which codes for a methyltransferase involved in two nonconsecutive methylation reactions during ubiquinol biosynthesis (Table S3). The UbiG methyltransferase is also rate limiting in ubiquinone biosynthesis (37). Furthermore, RedB also directly downregulates the expression of *atpA*, *atpG*, and *atpH*, which code for the alpha, gamma, and delta subunits of F_o_F_1_ ATP synthase (Table S2), and indirectly represses the expression of *atpB*, which encodes subunit A of F_o_ ATP synthase (Table S3) (38). Thus, RedB directly and indirectly represses the expression of the photosystem, the electron carrier components responsible for generating membrane potential, and ATPase, which utilizes this potential for ATP generation.

It should also be noted that the photosystem, components of the photosystem electron transport chain (cytochrome *bc*_1_, cytochrome *Y*, and ubiquinone), and ATPase are all located in an “intracytoplasmic” membrane (ICM) complex (Fig. 5). The ICM is not present when these cells do not synthesize a photosystem (39). In this regard, RedB also activates the expression of four genes involved in fatty acid degradation: the *rcc01546* gene, which encodes the long-chain fatty acid CoA ligase; two genes, *rcc00405* and *gcdH*, that code for the acyl-CoA dehydrogenase; and the *atoB1* gene, which encodes the acetyl-CoA acetyltransferase (Table S3). This expression pattern suggests that RedB not only limits the synthesis of the photosystem but also appears to promote the degradation of the ICM by the stimulation of fatty acid degradation.

### RedB has a role in limiting energy production from carbohydrate metabolism.

**FIG 6:**
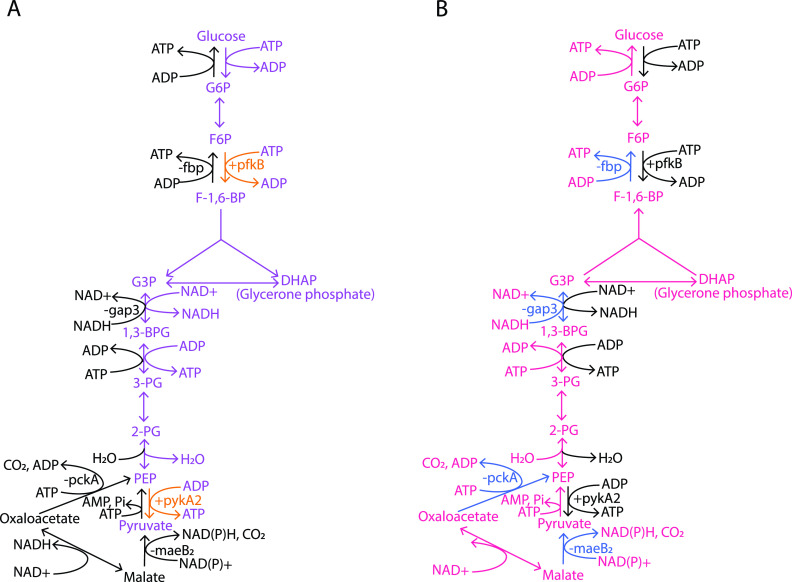
Steps in glycolysis and gluconeogenesis regulated by RedB. (A) The pathway of glycolysis is highlighted in violet. (B) The pathway of gluconeogenesis is highlighted in red. Individual steps in the pathway activated by RedB are indicated by + and also highlighted in orange. Steps repressed by RedB are highlighted in blue and indicated by −. G6P, glucose 6-phosphate; F6P, fructose 6-phosphate; F-1,6-BP, fructose 1,6-bisphosphate; G3P, glyceraldehyde 3-phosphate; DHAP, dihydroxyacetone phosphate/glycerone phosphate; 1,3-BPG, 1,3-bisphosphoglycerate; 3-PG, 3-phosphoglycerate; 2-PG, 2-phosphoglycerate; PEP, phosphoenolpyruvate.

Glycolysis and gluconeogenesis are two key pathways in carbohydrate metabolism that control the flow of carbon in divergent directions (Fig. 6) (40). In glycolysis, RedB directly activates pyruvate kinase, encoded by *pykA2* (Table S2), while indirectly activating 6-phosphofructokinase, encoded by *pfkB* (Fig. 6A and Table S3). These two enzymes catalyze two irreversible reactions of glycolysis, the phosphorylation of fructose-6-phosphate into fructose 1,6-bisphosphate and the dephosphorylation of PEP (phosphoenolpyruvate) into pyruvate, respectively (40). Conversely, RedB indirectly represses the expression of two enzymes catalyzing reverse reactions (the process of gluconeogenesis) at these similar steps (Fig. 6B and Table S3). These gluconeogenesis enzymes are fructose 1,6-bisphosphatase, encoded by *fbp*, and phosphoenolpyruvate carboxykinase, encoded by *pckA*. Additionally, RedB also indirectly downregulates the expression of malate dehydrogenase (*maeB2*), catalyzing the irreversible first step of gluconeogenesis, and also directly represses type I glyceraldehyde-3-phosphate dehydrogenase (*gap3*), catalyzing a reversible intermediate step in gluconeogenesis, namely, the dephosphorylation of 3-phospho-d-glyceroyl-phosphate into d-glyceraldehyde-3-phosphate (Tables S2 and S3) (40). Overall, it can be inferred that RedB favors glycolysis by increasing the expression of key enzymes in this pathway while also inhibiting sugar synthesis through the repression of enzymes involved in gluconeogenesis.

**FIG 7:**
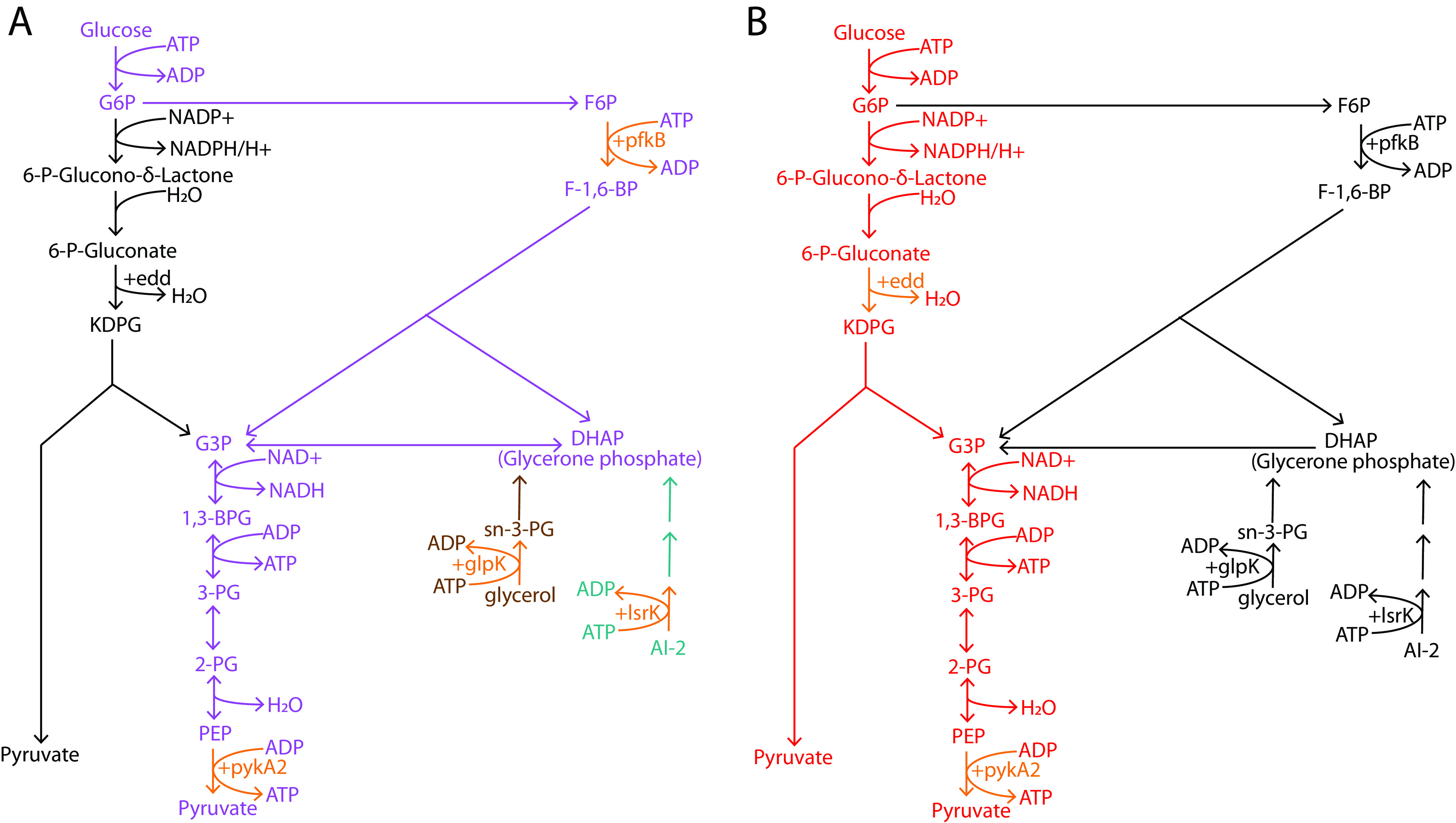
RedB regulation of glycolysis, the Entner-Doudoroff shunt, glycerol degradation, and autoinducer-2 (AI-2) degradation. (A) The pathway of glycolysis is highlighted in violet, the pathway of glycerol degradation is highlighted in brown, and the pathway of autoinducer-2 degradation is highlighted in dark green. (B) The pathway of the Entner-Doudoroff shunt is highlighted in red. Individual steps in the pathway activated by RedB are indicated by + and also highlighted in orange. KDPG, 2-keto-3-deoxy-6-phosphogluconate.

In line with RedB’s activation of glycolysis, RedB also directly upregulates *glpK*, which codes for the enzyme glycerol kinase that produces glycerol-3-phosphate, which is further converted into glycerone phosphate, another intermediate of glycolysis (Fig. 7A and Table S2) (41). Similarly, the degradation of autoinducer-2, a signaling molecule involved in quorum sensing (42), also generates glycerone phosphate as one of its end products. Specifically, RedB indirectly activates autoinducer-2 kinase (*lsrK*), which catalyzes the first step of autoinducer-2 degradation (Fig. 7A and Table S2).

The Entner-Doudoroff shunt is another interesting pathway that RedB appears to directly regulate. In this pathway, glucose is converted via two steps into 6-phosphogluconate, which is then catabolized by multiple steps into pyruvate (Fig. 7B) (41). In this regard, we observed that RedB directly activates the expression of *edd*, which encodes 6-phosphogluconate dehydrogenase, which catalyzes the first committed step of the pathway (Table S2). What is of interest in the activation of the Entner-Doudoroff shunt is that it generates less energy than does glycolysis. Specifically, one glucose molecule entering this pathway generates two molecules of pyruvate but only one ATP, one NADPH, and one NADH molecule (41). This is half the amount of energy production that glycolysis generates. Thus, as is the case with the repression of photosynthesis and ATPase, RedB appears to limit ATP production by activating the Entner-Doudoroff shunt.

Additionally, RedB stimulates carbon flow through the pentose phosphate pathway, which generates NADPH and several 5-carbon sugars such as ribose-5-phosphate, a precursor to nucleotides (43). For example, RedB directly activates the expression of *xylF*, which codes for a d-xylose ABC transporter substrate binding protein, potentially facilitating the import of d-xylose into the cytosol (Table S2). RedB also indirectly activates the expression of xylose isomerase (*xylA*), which catalyzes the isomerization of d-xylose to d-xylulose and directly activates the expression of xylulokinase (*xylB*), which catalyzes the phosphorylation of d-xylulose to form d-xylulose 5-phosphate, which is an intermediate in the pentose phosphate pathway (Table S3). Ribose is also a substrate in the pentose phosphate pathway, and in this regard, RedB indirectly activates the expression of *rbsC*, which encodes a high-affinity ribose transporter permease in the ABC transporter family (44).

**FIG 8:**
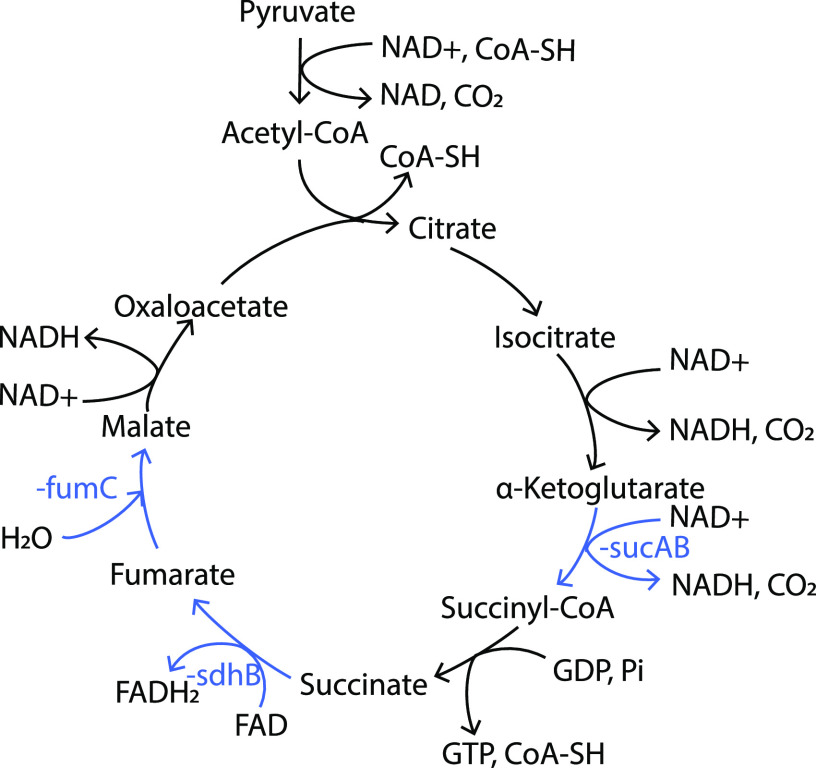
RegB regulation of the TCA cycle. Pyruvate feeding into the TCA cycle is highlighted in black. Genes coding for enzymes in this pathway that undergo repression by RedB are highlighted in blue. FAD, flavin adenine dinucleotide; FADH_2_, reduced flavin adenine dinucleotide. CoA-SH, coenzyme A.

Consistent with our conclusion that RedB has a role in limiting energy production, we also observed that RedB downregulates the expression of proteins involved in the tricarboxylic acid (TCA) cycle (Fig. 8). For example, RedB indirectly represses *sucA*, *sucB*, *sdhB*, and *fumC*, coding for 2-oxoglutarate (α-ketoglutarate) dehydrogenase, dihydrolipoyllysine succinyltransferase, succinate dehydrogenase, and fumarate hydratase, respectively (Table S3). Additionally, RedB inhibits several genes encoding a C_4_-dicarboxylate transporter, which is used mainly for transporting intermediates in the TCA cycle, such as malate, fumarate, and succinate (Fig. 8). Specifically, RedB indirectly represses three clustered genes, *dctP*, *dctQ*, and *dctM*, encoding the tripartite ATP-independent periplasmic (TRAP) transporter (the *dctP* gene codes for a C_4_-dicarboxylate binding protein, and the *dctQ* and *dctM* genes together encode a C_4_-dicarboxylate transporter) (Table S3) (45, 46). Similarly, a predicted sugar ABC transporter encoded by *rcc02022* is indirectly downregulated by RedB (Table S3). It is plausible that the transporter is used for transporting similar substrates such as malate, fumarate, and succinate. Collectively, the observed changes in the expression of genes involved in central metabolism indicate that RedB has a significant role in limiting cellular energy production.

### RedB suppresses *de novo* pyrimidine and purine biosynthesis.

**FIG 9:**
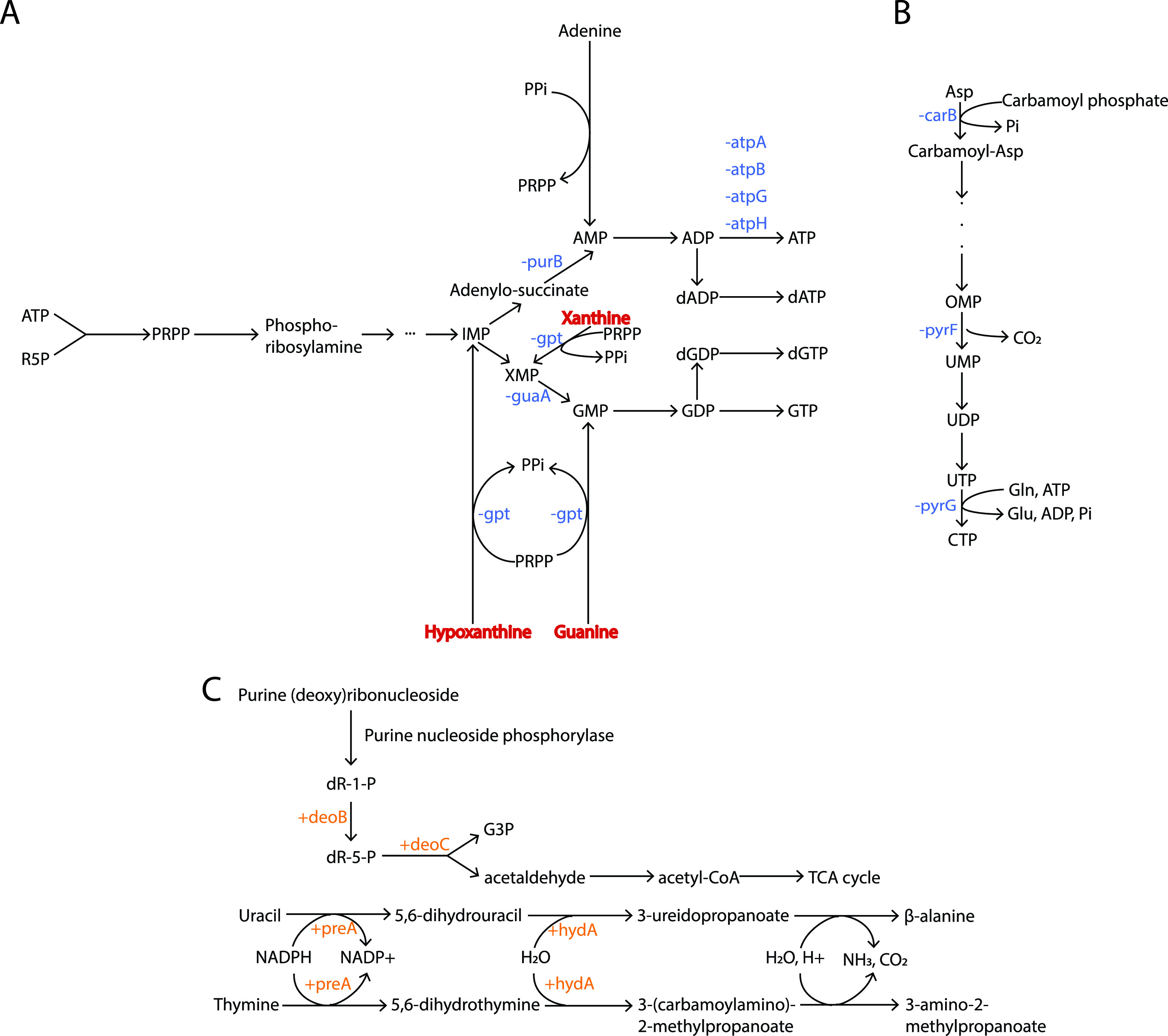
RedB regulation of nucleotide biosynthesis and degradation. (A) Pathway of purine nucleotide biosynthesis showing that RedB suppresses *de novo* ATP and GTP formation as well as the salvage of IMP, XMP, and GMP. R5P, ribose-5-phosphate. (B) Pathway of pyrimidine ribonucleotide *de novo* biosynthesis. (C) Pathways of nucleoside degradation regulated by RedB, including purine ribonucleoside degradation, purine deoxyribonucleoside degradation, uracil degradation, and thymine degradation. The activation of genes is indicated by + and highlighted in orange. The repression of genes is indicated by − and highlighted in blue. PRPP, phosphoribosyl diphosphate; OMP, orotidine 5’-monophosphate; dR-1-P, deoxyribose-1-phosphate; dR-5-P, deoxyribose-5-phosphate.

As discussed above, RedB directly and indirectly favors catabolic pathways that minimize substrate-level ATP formation, represses the synthesis of a photosystem that generates ATP, and also represses the synthesis of ATPase. It is therefore not surprising that RedB also directly and indirectly inhibits the *de novo* synthesis of ATP and GTP by indirectly downregulating the *purB* and *guaA* genes, which are involved in purine metabolism (Fig. 9A and Table S3). Specifically, *purB* codes for adenylosuccinate lyase, catalyzing the second step in the ATP branch of the purine biosynthesis pathway. The product of this reaction is AMP, which in later steps is converted into ADP and then ATP. The other gene repressed by RedB is *guaA*, which codes for GMP synthase in the second step of the GTP branch of the purine biosynthesis pathway. This enzyme catalyzes the conversion of XMP into GMP, with later steps converting GMP into GDP and then GTP. Regarding the purine salvage pathway, RedB also indirectly represses *gpt*, coding for a xanthine phosphoribosyltransferase that is involved in the formation of IMP, XMP, and GMP (Table S3). Thus, RedB not only suppresses substrate level and ATPase-mediated synthesis of ATP but also represses the *de novo* synthesis of ATP and GTP.

RedB also inhibits the biosynthesis of pyrimidine ribonucleotides by directly repressing the expression of *pyrF* (Table S2) and indirectly repressing the expression of two genes, *carB* and *pyrG* (Fig. 9B and Table S3). Among these genes, *carB* encodes the large subunit of the carbamoyl phosphate synthase that catalyzes the first step in *de novo* ribonucleotide biosynthesis, namely, the transamination reaction between glutamine and hydrogen carbonate to generate carbamoyl phosphate. The *pyrF* gene codes for a decarboxylase to catalyze decarboxylation from orotidine 5′-phosphate to UMP, which is an intermediate step in the pathway to generate UTP and CTP. Finally, the *pyrG* gene codes for the CTP synthetase to catalyze the last step, involving the conversion of UTP into CTP (Fig. 8B). This step is also shared by the salvage pathway of the ribonucleobase uracil.

In addition to suppressing pyrimidine nucleotide synthesis, RedB also stimulates pyrimidine degradation. Specifically, RedB directly upregulates *preA*, which encodes subunit B of the dihydropyrimidine dehydrogenase (Table S2), and indirectly upregulates *hydA*, which encodes the dihydropyrimidinase (Fig. 9C and Table S3). These enzymes are involved in uracil and thymine degradation. Beyond that, RedB stimulates degradation pathways for both pyrimidine and purine deoxynucleosides by activating two enzymes catalyzing two consecutive steps to degrade the remaining 2-deoxyribose-1-phosphate moiety into acetaldehyde and glyceraldehyde-3-phosphate. These two enzymes are encoded by the *deoB* and *deoC* genes, respectively (Fig. 9C), with the latter being directly upregulated by RedB (Tables S2 and S3). The resulting product, acetaldehyde, can be further converted into acetyl-CoA to enter the TCA cycle or fatty acid biosynthesis, whereas glyceraldehyde-3-phosphate is an intermediate of glycolysis. Overall, it can be inferred that RedB promotes both pyrimidine and purine nucleotide degradation.

### RedB represses protein synthesis.

**FIG 10:**
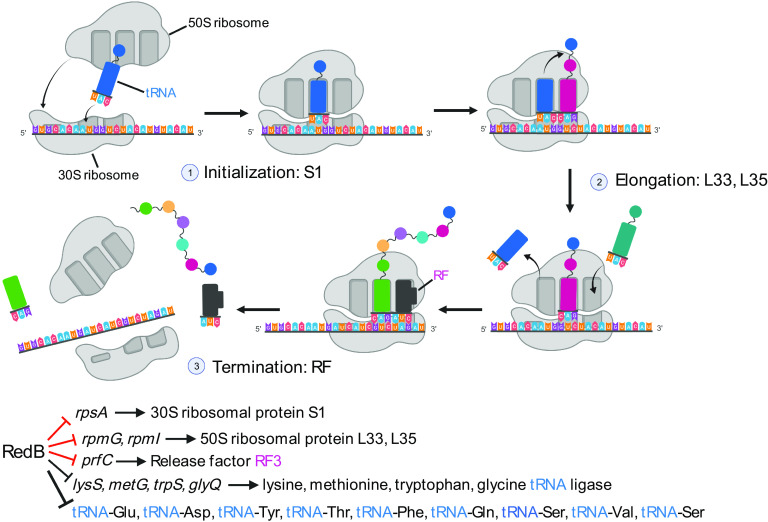
Schematic diagram of the bacterial protein synthesis process and typical genes or tRNA transcripts repressed by RedB. The schematic diagram shows the three stages of translation, which are initialization, elongation, and termination. In the diagram, the 50S ribosome, 30S ribosome, tRNA charged with amino acids, and release factor (RF) are indicated. Genes directly repressed by RedB are indicated with red inhibition signs. (Adapted from “protein translation,” by BioRender.com.)

Regarding translation, RedB directly represses three genes, *rpsA*, *rpmG*, and *rpmI*, encoding 30S ribosomal protein S1, 50S ribosomal protein L33, and 50S ribosomal protein L35, respectively (Fig. 10 and Table S2). RedB also directly represses the expression of *prfC*, encoding peptide chain release factor 3 (RF-3), which is involved in the elongation of peptide chains during translation. Of particular note is the 1.51-fold repression of S1 expression, given that S1 has a central role in promoting the initial binding and assembly of ribosomes into mRNA (47). Thus, the RedB-mediated repression of S1 synthesis could profoundly affect the suppression of overall cellular protein synthesis (Fig. 10).

RedB also indirectly represses the expression of four amino acid tRNA ligases, glycine-tRNA ligase, lysine-tRNA ligase, methionine-tRNA ligase, and tryptophan-tRNA ligase, which charge tRNAs with their cognate amino acids (Fig. 9 and Table S3). RedB also indirectly or directly downregulates the transcription of various tRNAs (Fig. 10 and Tables S2 and S3). In addition, RedB also indirectly represses the expression of a queuosine precursor transporter (encoded by *rcc03459*) (Table S3). Queuosine is a modified nucleoside at the first anticodon position of tRNAs for aspartic acid, asparagine, histidine, and tyrosine. This modification is thought to increase the strength of the codon-anticodon association (48). Therefore, it appears that RedB also inhibits translation by repressing the expression of tRNAs as well as the charging of several tRNAs with their cognate amino acids.

Regarding amino acid synthesis, RedB also represses the expression of enzymes in numerous amino acid biosynthetic pathways. For instance, RedB directly downregulates three genes, *rcc03447*, *serC*, and *serB*, encoding the enzymes phosphoglycerate dehydrogenase, phosphoserine aminotransferase, and phosphoserine phosphatase, respectively, which are involved in l-serine biosynthesis (Table S2). These enzymes convert 3-phosphoglycerate into serine sequentially through dehydrogenation, transamination, and dephosphorylation. Furthermore, RedB repression of l-serine synthesis also affects l-glycine levels, as l-serine is converted into l-glycine in a single step by serine hydroxymethyltransferase. *hisB*, encoding an imidazole glycerol-phosphate dehydratase, which catalyzes a dehydrolysis step in l-histidine biosynthesis, is another amino acid biosynthesis gene that is directly repressed by RedB (Table S2). Since this enzyme is unique to this pathway, it can be inferred that RedB also directly inhibits the biosynthesis of l-histidine.

The pathways leading to l-valine, l-leucine, and l-isoleucine biosynthesis share several enzymes that are repressed by RedB. Specifically, RedB indirectly represses the expression of both the *ilvN* and *ilvI* genes, which encode the small and large subunits of acetolactate synthase, respectively (Table S3). Acetolactate synthase actually catalyzes two different reactions, with one reaction leading to the synthesis of acetolactate, a precursor of both l-valine and l-leucine, and the other reaction leading to the synthesis of 2-aceto-2-hydroxybutanoate, a precursor of l-isoleucine. In addition, RedB also indirectly downregulates the *leuD* gene, encoding a 3-isopropylmalate dehydratase, which catalyzes a dehydrogenation reaction as well as a subsequent hydration reaction needed for l-leucine synthesis. l-Isoleucine biosynthesis requires l-threonine as an early substrate for its synthesis. Thus, it is not surprising that RedB also represses the biosynthesis of l-threonine from l-aspartate by indirectly downregulating the expression of *asd*, which codes for the aspartate-semialdehyde dehydrogenase. This enzyme catalyzes an intermediate dehydrogenation step in l-threonine biosynthesis. Finally, RedB also directly represses an aminotransferase encoded by *ilvE1* (Fig. 3B), which is involved in the last steps of l-isoleucine, l-valine, and l-leucine biosynthesis (Table S2). Overall, it appears that RedB has a significant role in repressing the synthesis of these branched-chain amino acids as well as the synthesis of l-threonine.

We also observed that RedB inhibits the biosynthesis of l-phenylalanine by indirectly downregulating the *aroH* gene, which encodes a chorismate mutase that is unique to the pathway that converts chorismate into prephenate (Table S3). RedB also indirectly downregulates the expression of two enzymes involved in l-arginine biosynthesis, the large subunit of carbamoyl phosphate synthase, encoded by *carB*, and argininosuccinate synthase, encoded by *argG*. In addition to reducing the levels of enzymes involved in l-arginine biosynthesis, RedB also favors the degradation of l-arginine. In this case, RedB indirectly activates two genes, *rocF* and *arcB2*, which code for enzymes involved in the degradation of l-arginine to l-proline, which is one of two pathways for l-proline biosynthesis. The other pathway synthesizes l-proline from l-glutamate, with RedB indirectly upregulating *proC*, which codes for the pyrroline-5-carboxylate reductase, which catalyzes the final step of proline formation. Why would RedB suppress the synthesis of many amino acids and yet apparently stimulate proline synthesis? Apart from being a component of proteins, proline also serves as an osmotic protectant in bacteria (49). Consequently, the enhancement of l-proline biosynthesis by RedB may serve to improve the resistance of bacteria to osmotic stress.

Finally, beyond the suppression of amino acid synthesis, RedB also appears to stimulate the degradation of amino acids. For example, RedB activates *kynU*, which encodes a kynureninase (Table S3) shared by two disparate pathways involved in l-tryptophan degradation. RedB also promotes l-serine degradation by indirectly upregulating the expression of *sdaA*, which codes for an ammonia-lyase (Table S3) involved in the degradation of serine to 2-aminoprop-2-enoate, the first and key step in l-serine degradation.

Overall, our transcriptome results indicate that RedB broadly suppresses protein synthesis by (i) repressing several ribosomal proteins, one of which is required for the formation of the translation preinitiation complex; (ii) suppressing the expression of several tRNAs as well as several tRNA ligases; (iii) suppressing the biosynthesis of l-serine, l-glycine, l-histidine, l-valine, l-leucine, l-isoleucine, l-threonine, l-phenylalanine, and l-arginine; and (iv) promoting the degradation of l-arginine, l-tryptophan, and l-serine.

### RedB’s role in nitrogen metabolism.

There are several means that a cell uses to obtain and recycle nitrogen. Regarding nitrogen fixation, a process that utilizes large amounts of ATP, RegB represses the expression of several genes that would impact this process. For example, RedB directly represses *dnaK*, which codes for a chaperone involved in the [2Fe-2S] iron-sulfur cluster pathway (Table S2). This enzyme complex functions mainly as a chaperone to help transfer a [2Fe-2S] iron-sulfur cluster to apoproteins (50). In nitrogenase, a [2Fe-2S] iron-sulfur cluster serves as a precursor of the iron-molybdenum (FeMo) cofactor, which constitutes the active site of molybdenum-containing nitrogenases (51). In this regard, RedB also indirectly downregulates *nifH*, which encodes not only a molybdenum chelatase that catalyzes the maturation reaction of the FeMo cofactor in NifDK but also a 4Fe-4S-containing reductase component of the nitrogenase complex (Table S3) (51). Therefore, we deduce that RedB likely inhibits nitrogenase maturation to repress nitrogen fixation. This may be a function of RegB’s reduction of protein synthesis (discussed above), which would reduce the cell’s need for fixed nitrogen.

Regarding nitrogen recycling, there are several reserves that a cell can use. For example, putrescine is a typical polyamine that arises from l-arginine or l-ornithine degradation that can be further degraded into 4-aminobutanoate (GABA) to serve as a nitrogen source in bacteria (52). During GABA degradation, GABA is converted in two steps into succinate, a substrate of the TCA cycle. Importantly, RedB indirectly activates the *gabT1* gene, encoding an enzyme that serves as both a glutamate decarboxylase and a γ-aminobutyrate aminotransferase to catalyze the first step of GABA degradation (Table S3).

Another amine rich in nitrogen is allantoin, a product of purine degradation. RedB also promotes allantoin degradation by indirectly activating the expression of *allA*, which codes for a ureidoglycolate lyase, an enzyme unique to this pathway, breaking down ureidoglycolate into glyoxylate and urea in the last step (Table S3).

### RedB’s role in sulfur metabolism and arsenate detoxification.

The biological incorporation of sulfur requires that sulfate must first be reduced into hydrogen sulfide, a process referred to as assimilatory sulfate reduction (53). In this regard, RedB indirectly represses all three enzymes involved in the entire pathway of assimilatory sulfate reduction (Table S3). Specifically, RedB indirectly downregulates the expression of *sat*, which encodes an adenylylsulfate kinase that catalyzes not only the activation of sulfate to adenosine 5-phosphosulfate (APS) but also the phosphorylation of APS to 3-phosphoadenylyl-sulfate (PAPS). In addition, RedB indirectly represses phosphoradenylyl-sulfate reductase (encoded by *cysH*), which catalyzes the reduction of PAPS to sulfite in the third step. In the last step, RedB downregulates sulfite reductase (*cysI*), which serves as a ferredoxin to shuttle the electrons needed to reduce sulfite into hydrogen sulfide. Thus, RedB’s inhibition of assimilatory sulfate reduction likely limits the ability of R. capsulatus to assimilate sulfate into the cell, which is needed for the biosynthesis of sulfur-containing compounds.

With regard to arsenate detoxification, RedB indirectly downregulates the synthesis of arsenate reductase (encoded by *rcc02458*), which catalyzes the key step in reducing arsenate to arsenite (Table S3). The neutral form of arsenite can then be exported out of the cell with the help of an aquaglyceroporin channel. Therefore, RedB inhibits arsenate detoxification. As an analog of phosphate, arsenate can hamper phosphorylation reactions, leading to the uncoupling of oxidative phosphorylation (54) to inhibit ATP generation. Thus, RedB’s inclination to prevent energy production is in line with its tendency to inhibit arsenate detoxification.

### Conclusions.

The CRP/FNR family of transcriptional regulators represents a large clade in which there are several subgroups. The best-characterized subgroup members are CRP and FNR, which typically activate the expression of a large number of genes necessary for carbohydrate and anaerobic metabolism, respectively (4). In this study, we report the first characterization of RedB, a CRP/FNR homolog involved in the anaerobic regulation of energy and redox potential. Sequence similarity network analysis of the CRP/FNR superfamily indicates that RedB is a member of a previously uncharacterized class of this superfamily that is distinct from both CRP and FNR (Fig. 1). Motivated by the novelty of this transcription factor, we sought to investigate its regulatory function in R. capsulatus.

**FIG 11:**
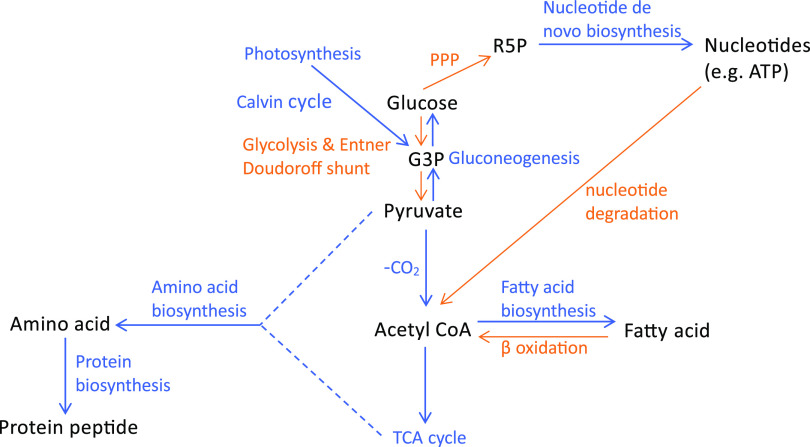
Summary of major metabolic and cellular activities regulated by the RedB regulon. Activities upregulated by RedB are highlighted in orange, whereas activities repressed by RedB are highlighted in blue. PPP, pentose phosphate pathway; R5P, ribose-5-phosphate; G3P, glyceraldehyde-3-phosphate.

Like CRP and FNR, we show that RedB is also a global regulator of gene expression. However, unlike CRP and FNR, which have roles in energy generation, RedB broadly functions to repress the expression of enzymes involved in multiple energy-generating pathways. Specifically, RedB impedes energy production by constraining pathways that produce large amounts of ATP and reducing equivalents via the suppression of the enzyme synthesis necessary for the TCA cycle that produces large amounts of cellular energy (Fig. 11). Concurrently, RedB induces the expression of enzymes that promote the flow of carbon through the Entner-Doudoroff shunt, which produces less energy than does glycolysis (Fig. 11). RedB also suppresses photosynthesis and limits *de novo* ATP synthesis. These roles are distinctly different from those of CRP and FNR, which enhance energy production. Additionally, RedB also ramps down protein synthesis by reducing the expression of ribosomal proteins involved in translation initiation, tRNA synthesis, tRNA charging, and amino acid biosynthesis and by promoting amino acid degradation. To our knowledge, RedB is the first example of a global transcription factor that has a role in limiting energy production. Consequently, future studies on the mechanism of redox control exhibited by RedB and analyses of the role of other members of the RedB subfamily are warranted.

Finally, in a companion article, we show that many genes regulated by RedB significantly overlap genes that constitute members of the FnrL regulon (the *Rhodobacter* ortholog of FNR) (28). Thus, RedB appears to have a major role in functioning as a damper to counter the energy-producing role of FnrL (28).

## MATERIALS AND METHODS

### Strains, media, and growth conditions.

Rhodobacter capsulatus wild-type strain SB1003 was used as the major material to construct a deletion strain, Δ*redB*, that removes codons 4 through 216 (out of a total of 218 codons). First, a fragment was amplified by combining 500 bp of the upstream and downstream regions of *redB* with 9 bp beginning from the start codon and 9 bp ending at the stop codon in the natural order (primers are shown in Table S5 in the supplemental material). The fragment was then ligated to a suicide plasmid, pZJD29 (55), which contains a gentamicin drug resistance gene and a counterselectable *sacA* gene. The resulting plasmid, pZJD29-Δ*redB*, was transformed into E. coli S17-1 and then conjugated into SB1003 to attain the Δ*redB* deletion strain, as described previously (56). Both the Δ*redB* and wild-type SB1003 strains were grown either on solidified peptone yeast extract (PY) agarose plates supplemented with MgCl_2_ and MgSO_4_ at a final concentration of 2 mM or in PY liquid medium (56). To collect the anaerobically grown cells, three biological replicates of both strains were first cultured in 5 mL PY liquid medium semiaerobically at 34°C with shaking at 200 rpm overnight. The cells were then subcultured into 8.5-mL screw-cap vials and grown anaerobically overnight at 34°C, with illumination provided from a 60-W incandescent light source. After that, the cells were transferred into new screw-cap vials, adjusted to an optical density (OD) adjusted to 0.03, and allowed to grow anaerobically until they reached an OD at 660 nm (OD_660_) of 0.3 for harvesting. A Unico 1100 RS spectrophotometer was used to check the OD in anaerobic vials.

### RNA isolation, validation, and sequencing (RNA-seq).

Cells at an OD_660_ of ~0.3 were immediately chilled in an ice-water bath to stop growth. Next, 2-mL aliquots from each biological replicate were transferred into Eppendorf tubes and centrifuged at 6,000 rpm for 3 min at 4°C. The cell pellets were collected for total RNA extraction in the following way. The pellets were first dissolved in 100 μL TE (10 mM Tris-HCl, 1 mM EDTA [pH 8.0]) buffer containing 10 mg/mL lysozyme, kept at room temperature for 3 min, and then treated with a Bioline isolate RNA extraction kit. One unit of Turbo DNase (Thermo Fisher) was added to the extracted sample, and the sample was incubated at 37°C for half an hour to remove DNA. The Zymogen Directzol RNA extraction kit was then applied to clean up the sample. Finally, quantitative reverse transcriptase PCR (qRT-PCR) was performed on the housekeeping gene *rpoZ* in the presence and absence of reverse transcriptase to check for DNA removal.

The extracted RNA samples were sent to the University of Wisconsin—Madison Biotechnology Center, where the quantity and integrity of the samples were verified using NanoDrop2000 and Agilent 2100 Bioanalyzer systems. RNAs meeting the requirements of Illumina sample input guidelines were prepared using the Illumina TruSeq stranded total RNA kit (Illumina Inc., San Diego, CA, USA), with minor revisions. Two micrograms of total RNA of each sample was treated using the RiboZero rRNA removal (bacteria) kit (Epicentre Inc., Madison, WI, USA) to remove the rRNA. The RNA samples were then fragmented by incubation with divalent cations under increasing temperatures. SuperScript II reverse transcriptase (Invitrogen, Carlsbad, CA, USA), random primers, and actinomycin D were added to the RNA samples to reverse transcribe the mRNA into first-strand cDNA. A second strand of cDNA was then synthesized utilizing second-strand marking master mix. After that, paramagnetic beads were harnessed to purify the resulting double-stranded DNA (dsDNA) with blunt ends. A single “A” (adenine) base was added to the 3′ ends of DNA products by incubating dsDNA with A-tailing mix, and the dsDNA was then ligated to adapters that have a single “T” (thymine) base overhang at their 3′ ends. dsDNAs ligated with adapters were further purified with paramagnetic beads and then amplified for 10 cycles through linker-mediated PCR (LM-PCR), using PCR master mix and a PCR primer cocktail. After that, the PCR products were cleaned up with paramagnetic beads. An Agilent DNA1000 chip (Agilent Technologies Inc., Santa Clara, CA, USA) was used to evaluate the quality of the built library, and a Qubit dsDNA HS assay kit (Invitrogen, Carlsbad, CA, USA) was used to measure the quantity of the library. After standardizing the DNA in the library to 2 μM, standard cluster kits (v3) and the Illumina cluster station were applied to the generation of clusters. Standard SBS chemistry (v3) was used for single 100-bp sequencing on an Illumina HiSeq2000 sequencer, and the standard Illumina pipeline, version 1.8.2, was used for the analysis of images.

For validation of the expression levels observed by RNA-seq, we performed qRT-PCR using primers designed to amplify select RT transcripts, as previously described by Kumka and Bauer (19). As shown in the companion article by Ke et al. (28), there are more highly significant divergent changes in gene expression between the Δ*fnrL* and Δ*redB* deletion strains than between Δ*redB* and wild-type cells. Thus, comparative qRT-PCR validation analysis of the Δ*redB* and Δ*fnrL* strains was undertaken to validate the observed divergent changes in transcript levels.

### ChIP strain construction.

To construct 3×FLAG-tagged RedB, an oligonucleotide containing the 3×FLAG sequence was fused to the last 21 nucleotides of the *redB* gene before the stop codon to serve as a reverse primer. 3×FLAG-tagged *redB* was then PCR amplified and inserted into the pSRKGm plasmid (57) between the NdeI and XbaI restriction sites. After that, the recombinant plasmid was conjugated to the Δ*redB* strain for ChIP-seq experiments. This plasmid was used as it provides tunable low-level (native) expression in R. capsulatus, as described previously (58–60).

### ChIP-seq.

Three biological replicates of the 3×FLAG-tagged RedB construct were grown anaerobically under the same conditions as the ones described above for the Δ*redB* strain but with a cumulative culture volume of 50 mL per sample. When the OD_660_ of a 50-mL culture reached 0.3, 1% formaldehyde was added, and the mixture was incubated for 20 min at room temperature to cross-link DNAs with proteins. Cross-linking was quenched by adding glycine to a final concentration of 0.125 M, and the mixture was incubated for 5 min. After centrifugation, the cell pellets were washed twice with chilled Tris-buffered saline (TBS) (50 mM Tris-HCl [pH 8.0], 150 mM NaCl) and then resuspended in 4 mL cold FA-M2 buffer (50 mM Tris-HCl [pH 8.0], 150 mM NaCl, 1 mM EDTA, 1% Triton X-100). The cells were then passed through French press twice at 18,000 lb/in^2^ and further sonicated 6 times for 10 s via needle sonication. Fifty microliters of the lysate of each sample was saved without immunoprecipitation, which later served as the input DNA after purification. The remaining cell lysates were immunoprecipitated by agitation with 100 μL anti-FLAG M2 affinity gel overnight at 4°C. The beads were centrifuged at 3,000 rpm for 1 min to remove the supernatant and then washed five times with 1 mL FA-M2 buffer for 15 min. After that, the beads were washed three more times with 1 mL 1× TBS for 15 min. Cross-linking was reversed by incubating the beads with 200 μL elution buffer (50 mM Tris-HCl [pH 8.0], 10 mM EDTA, 1% SDS) at 65°C for 30 min. The supernatants of both the immunoprecipitated samples and the input samples obtained as described above were incubated with 5 μL of 20 mg/mL proteinase K at 65°C overnight to remove the protein. DNAs were further purified using a phenol-chloroform DNA extraction method and washed with cold 75% ethanol. The DNA pellets were dried and then eluted in 20 μL nuclease-free water.

Like RNA sequencing, libraries were constructed and sequenced by the University of Wisconsin—Madison Biotechnology Center DNA Sequencing Facility. Both the input DNA and immunoprecipitated DNA were processed using a TruSeq ChIP sample preparation kit (Illumina), with minor modifications. Using solid phase reverse immobilization (SBIR)-based bead selection, libraries were size selected for an average of 350 bp. Single-end 100-bp sequencing was carried out on an Illumina HiSeq2000 sequencer.

### Data preprocessing, computer software, and data analysis for RNA sequencing and ChIP sequencing.

For both RNA-seq and ChIP-seq results, data were trimmed using the Trimmomatic program (61), with a sliding window of 5:25 and a minimum length of 40 bp, and raw counts of genes were produced with the HTSeq-count program. Specifically, for RNA-seq raw counts, the DESeq2 package in R was used to give rise to differentially expressed genes (62, 63). For analysis of ChIP-seq results, MACS (64) was utilized to decide on significantly enriched sites, with the binding sequence queried using MEME (65) with standard parameters, except that the width of the motif was set to be between 12 bp and 18 bp.

### Construction and analysis of the CRP/FNR sequence similarity network.

To generate the CRP/FNR protein sequence similarity network (SSN), we assembled a list of protein sequences from the InterPro database that contained a single N-terminal cyclic nucleotide binding domain (IPR000595) fused to a C-terminal CRP-type helix-turn-helix domain (IPR0012318) (20). This list was further curated to include only proteins of 190 to 280 residues long, as sequences outside this range were scarce. The curated list of 97,535 protein identifiers was inputted into the Enzyme Function Initiative-Enzyme Similarity Tool (EFI-EST) (21) to generate the SSN. Cytoscape 3.8.2 (66) was used for network analysis and the generation of figures. All networks used for analysis and in the figures are 50% representative node networks, meaning that each node represents a packet of proteins that are at least 50% identical to one another in sequence. This was done to limit the size of the network for ease of analysis, as large networks require significant computational resources. UniProt accession numbers and references for previously characterized CRP/FNR proteins used for SSN figures are shown in Table S1.

### Data availability.

The raw DNA read files for RNA-seq and ChIP-seq analyses were deposited in the Sequence Read Archive (SRA) under accession number PRJNA657631.

## References

[1] Matsui M, Tomita M, Kanai A. 2013. Comprehensive computational analysis of bacterial Crp/FNR superfamily and its target motifs reveals stepwise evolution of transcriptional networks. Genome Biol Evol 5:267–282. doi:10.1093/gbe/evt004. 23315382 PMC3590769

[2] Spiro S. 1994. The FNR family of transcriptional regulators. Antonie Van Leeuwenhoek 66:23–36. doi:10.1007/BF00871630. 7747934

[3] Zhou AF, Chen YI, Zane GM, He ZL, Hemme CL, Joachimiak MP, Baumohl JK, He Q, Fields MW, Arkin AP, Wall JD, Hazen TC, Zhou JZ. 2012. Functional characterization of Crp/Fnr-type global transcriptional regulators in *Desulfovibrio vulgaris* Hildenborough. Appl Environ Microbiol 78:1168–1177. doi:10.1128/AEM.05666-11. 22156435 PMC3273024

[4] Korner H, Sofia HJ, Zumft WG. 2003. Phylogeny of the bacterial superfamily of Crp-FNR transcription regulators: exploiting the metabolic spectrum by controlling alternative gene programs. FEMS Microbiol Rev 27:559–592. doi:10.1016/S0168-6445(03)00066-4. 14638413

[5] Sharma H, Yu SN, Kong JL, Wang JM, Steitz TA. 2009. Structure of apo-CAP reveals that large conformational changes are necessary for DNA binding. Proc Natl Acad Sci USA 106:16604–16609. doi:10.1073/pnas.0908380106. 19805344 PMC2745332

[6] Won HS, Yamazaki T, Lee TW, Yoon MK, Park SH, Kyogoku Y, Lee BJ. 2000. Structural understanding of the allosteric conformational change of cyclic AMP receptor protein by cyclic AMP binding. Biochemistry 39:13953–13962. doi:10.1021/bi000012x. 11076538

[7] Zheng DL, Constantinidou C, Hobman JL, Minchin SD. 2004. Identification of the CRP regulon using *in vitro* and *in vivo* transcriptional profiling. Nucleic Acids Res 32:5874–5893. doi:10.1093/nar/gkh908. 15520470 PMC528793

[8] Khoroshilova N, Beinert H, Kiley PJ. 1995. Association of a polynuclear iron-sulfur center with a mutant FNR protein enhances DNA binding. Proc Natl Acad Sci USA 92:2499–2503. doi:10.1073/pnas.92.7.2499. 7708673 PMC42245

[9] Fleischhacker AS, Kiley PJ. 2011. Iron-containing transcription factors and their roles as sensors. Curr Opin Chem Biol 15:335–341. doi:10.1016/j.cbpa.2011.01.006. 21292540 PMC3074041

[10] Kiley PJ, Beinert H. 1998. Oxygen sensing by the global regulator, FNR: the role of the iron-sulfur cluster. FEMS Microbiol Rev 22:341–352. doi:10.1111/j.1574-6976.1998.tb00375.x. 9990723

[11] Khoroshilova N, Popescu C, Munck E, Beinert H, Kiley PJ. 1997. Iron-sulfur cluster disassembly in the FNR protein of Escherichia coli by O-2: [4Fe-4S] to [2Fe-2S] conversion with loss of biological activity. Proc Natl Acad Sci USA 94:6087–6092. doi:10.1073/pnas.94.12.6087. 9177174 PMC21006

[12] Myers KS, Yan H, Ong IM, Chung D, Liang K, Tran F, Keleş S, Landick R, Kiley PJ. 2013. Genome-scale analysis of *Escherichia coli* FNR reveals complex features of transcription factor binding. PLoS Genet 9:e1003565. doi:10.1371/journal.pgen.1003565. 23818864 PMC3688515

[13] Akhter Y, Tundup S, Hasnain SE. 2007. Novel biochemical properties of a CRP/FNR family transcription factor from *Mycobacterium tuberculosis*. Int J Med Microbiol 297:451–457. doi:10.1016/j.ijmm.2007.04.009. 17702648

[14] Shelver D, Kerby RL, He YP, Roberts GP. 1997. CooA, a CO-sensing transcription factor from *Rhodospirillum rubrum*, is a CO-binding heme protein. Proc Natl Acad Sci USA 94:11216–11220. doi:10.1073/pnas.94.21.11216. 9326589 PMC23420

[15] He YP, Shelver D, Kerby RL, Roberts GP. 1996. Characterization of a CO-responsive transcriptional activator from *Rhodospirillum rubrum*. J Biol Chem 271:120–123. doi:10.1074/jbc.271.1.120. 8550545

[16] Ferguson SJ, Jackson JB, McEwan AG. 1987. Anaerobic respiration in the Rhodospirillaceae: characterisation of pathways and evaluation of roles in redox balancing during photosynthesis. FEMS Microbial Rev 46:117–143. doi:10.1111/j.1574-6968.1987.tb02455.x.

[17] Tichi MA, Tabita FR. 2001. Interactive control of *Rhodobacter capsulatus* redox-balancing systems during phototrophic metabolism. J Bacteriol 183:6344–6354. doi:10.1128/JB.183.21.6344-6354.2001. 11591679 PMC100130

[18] Kumka JE, Schindel H, Fang M, Zappa S, Bauer CE. 2017. Transcriptomic analysis of aerobic respiratory and anaerobic photosynthetic states in *Rhodobacter capsulatus* and their modulation by global redox regulators RegA, FnrL and CrtJ. Microb Genom 3:e000125. doi:10.1099/mgen.0.000125. 29114403 PMC5643017

[19] Kumka JE, Bauer CE. 2015. Analysis of the FnrL regulon in *Rhodobacter capsulatus* reveals limited regulon overlap with orthologues from *Rhodobacter sphaeroides* and *Escherichia coli*. BMC Genomics 16:895. doi:10.1186/s12864-015-2162-4. 26537891 PMC4634722

[20] Blum M, Chang HY, Chuguransky S, Grego T, Kandasaamy S, Mitchell A, Nuka G, Paysan-Lafosse T, Qureshi M, Raj S, Richardson L, Salazar GA, Williams L, Bork P, Bridge A, Gough J, Haft DH, Letunic I, Marchler-Bauer A, Mi HY, Natale DA, Necci M, Orengo CA, Pandurangan AP, Rivoire C, Sigrist CJA, Sillitoe I, Thanki N, Thomas PD, Tosatto SCE, Wu CH, Bateman A, Finn RD. 2021. The InterPro protein families and domains database: 20 years on. Nucleic Acids Res 49:D344–D354. doi:10.1093/nar/gkaa977. 33156333 PMC7778928

[21] Gerlt JA, Bouvier JT, Davidson DB, Imker HJ, Sadkhin B, Slater DR, Whalen KL. 2015. Enzyme Function Initiative-Enzyme Similarity Tool (EFI-EST): a Web tool for generating protein sequence similarity networks. Biochim Biophys Acta 1854:1019–1037. doi:10.1016/j.bbapap.2015.04.015. 25900361 PMC4457552

[22] Strnad H, Lapidus A, Paces J, Ulbrich P, Vlcek C, Paces V, Haselkorn R. 2010. Complete genome sequence of the photosynthetic purple nonsulfur bacterium *Rhodobacter capsulatus* SB1003. J Bacteriol 192:3545–3546. doi:10.1128/JB.00366-10. 20418398 PMC2897665

[23] Holmquist PC, Holmquist GP, Summers ML. 2011. Comparing binding site information to binding affinity reveals that Crp/DNA complexes have several distinct binding conformers. Nucleic Acids Res 39:6813–6824. doi:10.1093/nar/gkr369. 21586590 PMC3159480

[24] Henry KK, Ross W, Myers KS, Lemmer KC, Vera JM, Landick R, Donohue TJ, Gourse RL. 2020. A majority of *Rhodobacter sphaeroides* promoters lack a crucial RNA polymerase recognition feature, enabling coordinated transcription activation. Proc Natl Acad Sci USA 117:29658–29668. doi:10.1073/pnas.2010087117. 33168725 PMC7703639

[25] Dischert V, Vignais PM, Colbeau A. 1999. The synthesis of Rhodobacter capsulatus HupSL hydrogenase is regulated by the two-component HupT/HupR system. Mol Microbiol 34:995–1006. doi:10.1046/j.1365-2958.1999.01660.x. 10594824

[26] Tokito MK, Daldal F. 1992. PetR, located upstream of the *fbcFBC* operon encoding the cytochrome *bc*_1_ complex, is homologous to bacterial response regulators and necessary for photosynthetic and respiratory growth of *Rhodobacter capsulatus*. Mol Microbiol 6:1645–1654. doi:10.1111/j.1365-2958.1992.tb00889.x. 1323023

[27] Mouncey NJ, Kaplan S. 1998. Cascade regulation of dimethyl sulfoxide reductase (*dor*) gene expression in the facultative phototroph *Rhodobacter sphaeroides* 2.4.1^T^. J Bacteriol 180:2924–2930. doi:10.1128/JB.180.11.2924-2930.1998. 9603883 PMC107260

[28] Ke N, Kumka JE, Fang M, Weaver B, Burstyn JN, Bauer CE. 2022. Redox brake regulator RedB and FnrL function as yin-yang regulators of anaerobic-aerobic metabolism in *Rhodobacter capsulatus*. Microbiol Spectr 10:e02354-22. doi:10.1128/Spectrum02354-22. 36106752 PMC9603517

[29] McCrindle SL, Kappler U, McEwan AG. 2005. Microbial dimethylsulfoxide and trimethylamine-N-oxide respiration. Adv Microb Physiol 50:147–198. doi:10.1016/S0065-2911(05)50004-3. 16221580

[30] Mangum JH, Magni G, Stadtman ER. 1973. Regulation of glutamine synthetase adenylylation and deadenylylation by enzymatic uridylylation and deuridylylation of PII regulatory protein. Arch Biochem Biophys 158:514–525. doi:10.1016/0003-9861(73)90543-2. 4150122

[31] van Heeswijk WC, Rabenberg M, Westerhoff HV, Kahn D. 1993. The genes of the glutamine-synthetase adenylylation cascade are not regulated by nitrogen in *Escherichia coli*. Mol Microbiol 9:443–457. doi:10.1111/j.1365-2958.1993.tb01706.x. 8412694

[32] Youvan DC, Ismail S. 1985. Light-harvesting II (B800-B850 complex) structural genes from *Rhodopseudomonas capsulata*. Proc Natl Acad Sci USA 82:58–62. doi:10.1073/pnas.82.1.58. 16593533 PMC396970

[33] Youvan DC, Bylina EJ, Alberti M, Begusch H, Hearst JE. 1984. Nucleotide and deduced polypeptide sequences of the photosynthetic reaction-center, B870 antenna, and flanking polypeptides from *R. capsulata*. Cell 37:949–957. doi:10.1016/0092-8674(84)90429-X. 6744416

[34] Gray KA, Daldal F. 1995. Mutational studies of the cytochrome *bc*_1_ complexes, p 747–774. *In* Blankenship RE, Madigan MT, Bauer CE (ed), Anoxygenic photosynthetic bacteria. Advances in photosynthesis and respiration, vol 2. Kluwer Academic Publishers, Dordrecht, The Netherlands.

[35] Verderber E, Lucast LJ, Van Dehy JA, Cozart P, Etter JB, Best EA. 1997. Role of the *hemA* gene product and delta-aminolevulinic acid in regulation of *Escherichia coli* heme synthesis. J Bacteriol 179:4583–4590. doi:10.1128/jb.179.14.4583-4590.1997. 9226269 PMC179295

[36] Beale SI. 1995. Biosynthesis and structures of porphyrins and hemes, p 153–177. *In* Blankenship RE, Madigan MT, Bauer CE (ed), Anoxygenic photosynthetic bacteria. Advances in photosynthesis and respiration, vol 2. Kluwer Academic Publishers, Dordrecht, The Netherlands.

[37] Lee SQE, Tan TS, Kawamukai M, Chen ES. 2017. Cellular factories for coenzyme Q(10) production. Microb Cell Fact 16:39. doi:10.1186/s12934-017-0646-4. 28253886 PMC5335738

[38] Borghese R, Crimi M, Fava L, Melandri BA. 1998. The ATP synthase *atpHAGDC* (F-1) operon from *Rhodobacter capsulatus*. J Bacteriol 180:416–421. doi:10.1128/JB.180.2.416-421.1998. 9440534 PMC106900

[39] Drews G, Golecki JR. 1995. Structure, molecular organization and biosynthesis of membranes of purple bacteria, p 231–257. *In* Blankenship RE, Madigan MT, Bauer CE (ed), Anoxygenic photosynthetic bacteria. Advances in photosynthesis and respiration, vol 2. Kluwer Academic Publishers, Dordrecht, The Netherlands.

[40] Gupta R, Gupta N. 2021. Glycolysis and gluconeogenesis, p 267–285. *In* Gupta R, Gupta N (ed), Fundamentals of bacterial physiology and metabolism. Springer, Singapore.

[41] Flamholz A, Noor E, Bar-Even A, Liebermeister W, Milo R. 2013. Glycolytic strategy as a tradeoff between energy yield and protein cost. Proc Natl Acad Sci USA 110:10039–10044. doi:10.1073/pnas.1215283110. 23630264 PMC3683749

[42] Pereira CS, Thompson JA, Xavier KB. 2013. AI-2-mediated signalling in bacteria. FEMS Microbiol Rev 37:156–181. doi:10.1111/j.1574-6976.2012.00345.x. 22712853

[43] Stincone A, Prigione A, Cramer T, Wamelink MMC, Campbell K, Cheung E, Olin-Sandoval V, Gruning N-M, Kruger A, Alam MT, Keller MA, Breitenbach M, Brindle KM, Rabinowitz JD, Ralser M. 2015. The return of metabolism: biochemistry and physiology of the pentose phosphate pathway. Biol Rev Camb Philos Soc 90:927–963. doi:10.1111/brv.12140. 25243985 PMC4470864

[44] Park Y, Park C. 1999. Topology of RbsC, a membrane component of the ribose transporter, belonging to the AraH superfamily. J Bacteriol 181:1039–1042. doi:10.1128/JB.181.3.1039-1042.1999. 9922273 PMC93476

[45] Forward JA, Behrendt MC, Wyborn NR, Cross R, Kelly DJ. 1997. TRAP transporters: a new family of periplasmic solute transport systems encoded by the *dctPQM* genes of *Rhodobacter capsulatus* and by homologs in diverse Gram-negative bacteria. J Bacteriol 179:5482–5493. doi:10.1128/jb.179.17.5482-5493.1997. 9287004 PMC179420

[46] Valentini M, Storelli N, Lapouge K. 2011. Identification of C_4_-dicarboxylate transport systems in *Pseudomonas aeruginosa* PAO1. J Bacteriol 193:4307–4316. doi:10.1128/JB.05074-11. 21725012 PMC3165536

[47] Sorensen MA, Fricke J, Pedersen S. 1998. Ribosomal protein S1 is required for translation of most, if not all, natural mRNAs in *Escherichia coli in vivo*. J Mol Biol 280:561–569. doi:10.1006/jmbi.1998.1909. 9677288

[48] Morris RC, Brown KG, Elliott MS. 1999. The effect of queuosine on tRNA structure and function. J Biomol Struct Dyn 16:757–774. doi:10.1080/07391102.1999.10508291. 10217448

[49] Abraham E, Rigo G, Szekely G, Nagy R, Koncz C, Szabados L. 2003. Light-dependent induction of proline biosynthesis by abscisic acid and salt stress is inhibited by brassinosteroid in *Arabidopsis*. Plant Mol Biol 51:363–372. doi:10.1023/a:1022043000516. 12602867

[50] Buren S, Jimenez-Vicente E, Echavarri-Erasun C, Rubio LM. 2020. Biosynthesis of nitrogenase cofactors. Chem Rev 120:4921–4968. doi:10.1021/acs.chemrev.9b00489. 31975585 PMC7318056

[51] Kim JS, Rees DC. 1992. Crystallographic structure and functional implications of the nitrogenase molybdenum iron protein from *Azotobacter vinelandii*. Nature 360:553–560. doi:10.1038/360553a0. 25989647

[52] Ferson AE, Wray LV, Fisher SH. 1996. Expression of the *Bacillus subtilis gabP* gene is regulated independently in response to nitrogen and amino acid availability. Mol Microbiol 22:693–701. doi:10.1046/j.1365-2958.1996.d01-1720.x. 8951816

[53] Sander J, Dahl C. 2009. Metabolism of inorganic sulfur compounds in purple bacteria, p 595–622. *In* Hunter CN, Daldal F, Thurnauer MC, Beatty JT (ed), The purple phototrophic bacteria. Advances in photosynthesis and respiration, vol 28. Springer, Dordrecht, The Netherlands.

[54] Bhuvaneswaran C. 1979. Influence of phosphorylation state ratio on energy-conservation in mitochondria treated with inorganic arsenate. Biochem Biophys Res Commun 90:1201–1206. doi:10.1016/0006-291X(79)91164-1. 518594

[55] Masuda S, Bauer CE. 2004. Null mutation of HvrA compensates for loss of an essential *relA*/*spoT*-like gene in *Rhodobacter capsulatus*. J Bacteriol 186:235–239. doi:10.1128/JB.186.1.235-239.2004. 14679243 PMC303453

[56] Young DA, Bauer CE, Williams JC, Marrs BL. 1989. Genetic evidence for superoperonal organization of genes for photosynthetic pigments and pigment-binding proteins in *Rhodobacter capsulatus*. Mol Gen Genet 218:1–12. doi:10.1007/BF00330558. 2550757

[57] Khan SR, Gaines J, Roop RM, II, Farrand SK. 2008. Broad-host-range expression vectors with tightly regulated promoters and their use to examine the influence of TraR and TraM expression on Ti plasmid quorum sensing. Appl Environ Microbiol 74:5053–5062. doi:10.1128/AEM.01098-08. 18606801 PMC2519271

[58] Fang M, Bauer CE. 2017. The vitamin B_12_-dependent photoreceptor AerR relieves photosystem gene repression by extending the interaction of CrtJ with photosystem promoters. mBio 8:e00261-17. doi:10.1128/mBio.00261-17. 28325764 PMC5362033

[59] Yamamoto H, Fang M, Dragnea V, Bauer CE. 2018. Differing isoforms of the cobalamin binding photoreceptor AerR oppositely regulate photosystem expression. Elife 7:e39028. doi:10.7554/eLife.39028. 30281022 PMC6199135

[60] Schindel HS, Bauer CE. 2016. The RegA regulon exhibits variability in response to altered growth conditions and differs markedly between *Rhodobacter* species. Microb Genom 2:e000081. doi:10.1099/mgen.0.000081. 28348828 PMC5359404

[61] Bolger AM, Lohse M, Usadel B. 2014. Trimmomatic: a flexible trimmer for Illumina sequence data. Bioinformatics 30:2114–2120. doi:10.1093/bioinformatics/btu170. 24695404 PMC4103590

[62] Robles JA, Qureshi SE, Stephen SJ, Wilson SR, Burden CJ, Taylor JM. 2012. Efficient experimental design and analysis strategies for the detection of differential expression using RNA-sequencing. BMC Genomics 13:484. doi:10.1186/1471-2164-13-484. 22985019 PMC3560154

[63] Love MI, Huber W, Anders S. 2014. Moderated estimation of fold change and dispersion for RNA-seq data with DESeq2. Genome Biol 15:550. doi:10.1186/s13059-014-0550-8. 25516281 PMC4302049

[64] Zhang Y, Liu T, Meyer CA, Eeckhoute J, Johnson DS, Bernstein BE, Nusbaum C, Myers RM, Brown M, Li W, Liu XS. 2008. Model-based analysis of ChIP-Seq (MACS). Genome Biol 9:R137. doi:10.1186/gb-2008-9-9-r137. 18798982 PMC2592715

[65] Bailey TL, Boden M, Buske FA, Frith M, Grant CE, Clementi L, Ren J, Li WW, Noble WS. 2009. MEME SUITE: tools for motif discovery and searching. Nucleic Acids Res 37:W202–W208. doi:10.1093/nar/gkp335. 19458158 PMC2703892

[66] Shannon P, Markiel A, Ozier O, Baliga NS, Wang JT, Ramage D, Amin N, Schwikowski B, Ideker T. 2003. Cytoscape: a software environment for integrated models of biomolecular interaction networks. Genome Res 13:2498–2504. doi:10.1101/gr.1239303. 14597658 PMC403769

