# Supplementary material for: RedB, a Member of the CRP/FNR Family, Functions as a Transcriptional Redox Brake
Source: Microbiol Spectr. 2022 Sep 15;10(5):e02353-22. doi: 10.1128/spectrum.02353-22 (PMC9603854; doi:10.1128/spectrum.02353-22)
Supplement: Supplemental file 1 — Supplemental material. Download spectrum.02353-22-s0004.pdf, PDF file, 3.8 MB [file spectrum.02353-22-s0004.pdf]

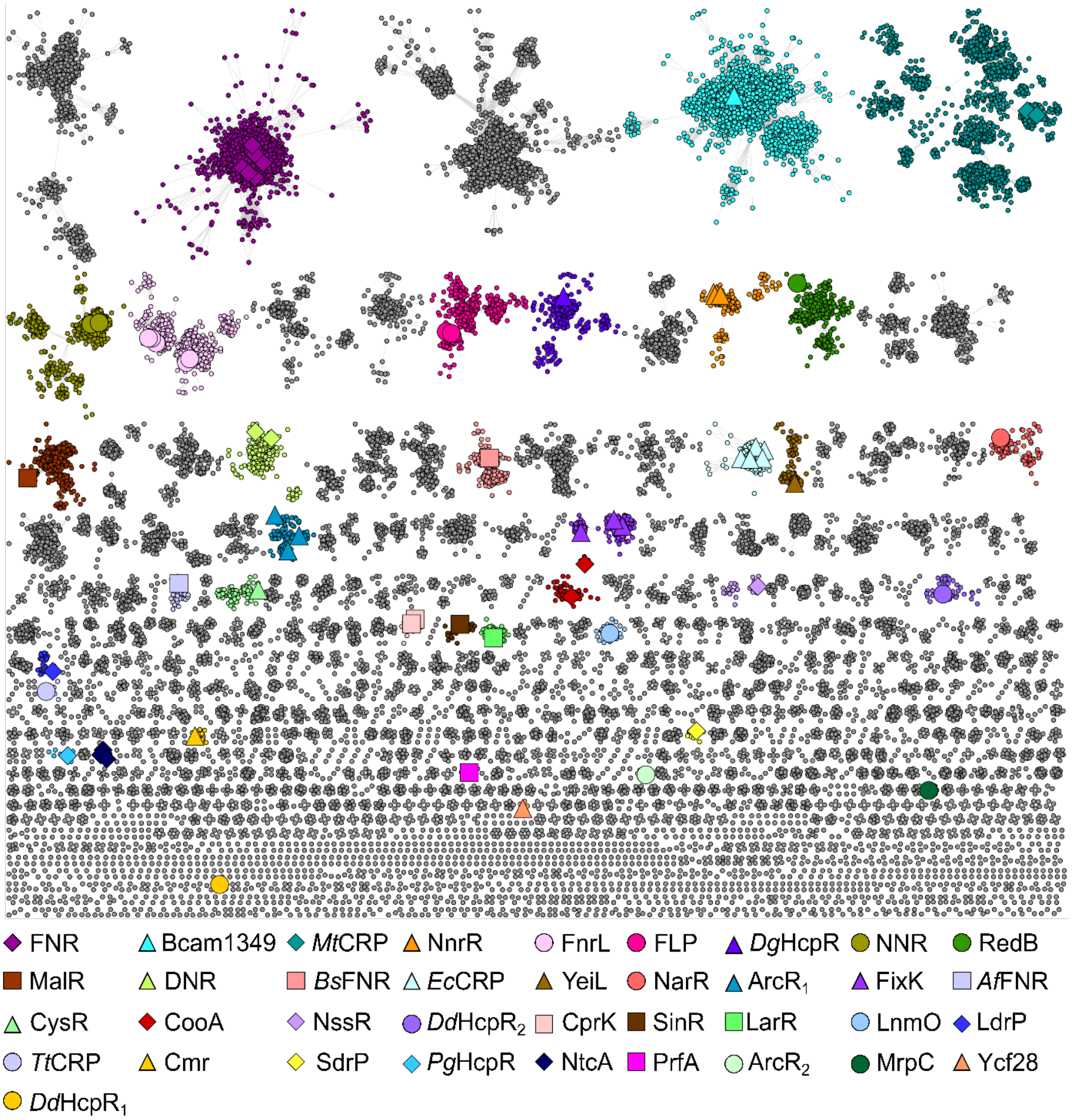

Supplementary Figure S1. A 50% representative sequence similarity network of the CRP/FNR superfamily with isofunctional clustering at an alignment score cutoff of 49. Clusters containing a characterized CRP/FNR transcription factor are colored according to their specified labels. Isofunctional clustering was achieved when the alignment score cutoff was raised to the point that all characterized CRP/FNR proteins with unique regulatory functions were completely isolated from one another. This process enables identification of protein clusters with similar regulatory functions. The large number of clusters within the network indicates the wide diversity of the CRP/FNR superfamily. Further, many clusters are gray, indicating that the function of the majority of these proteins is entirely unknown. A list of characterized CRP/FNR proteins that were mapped onto this sequence similarity network is provided in Supplementary Table S1.

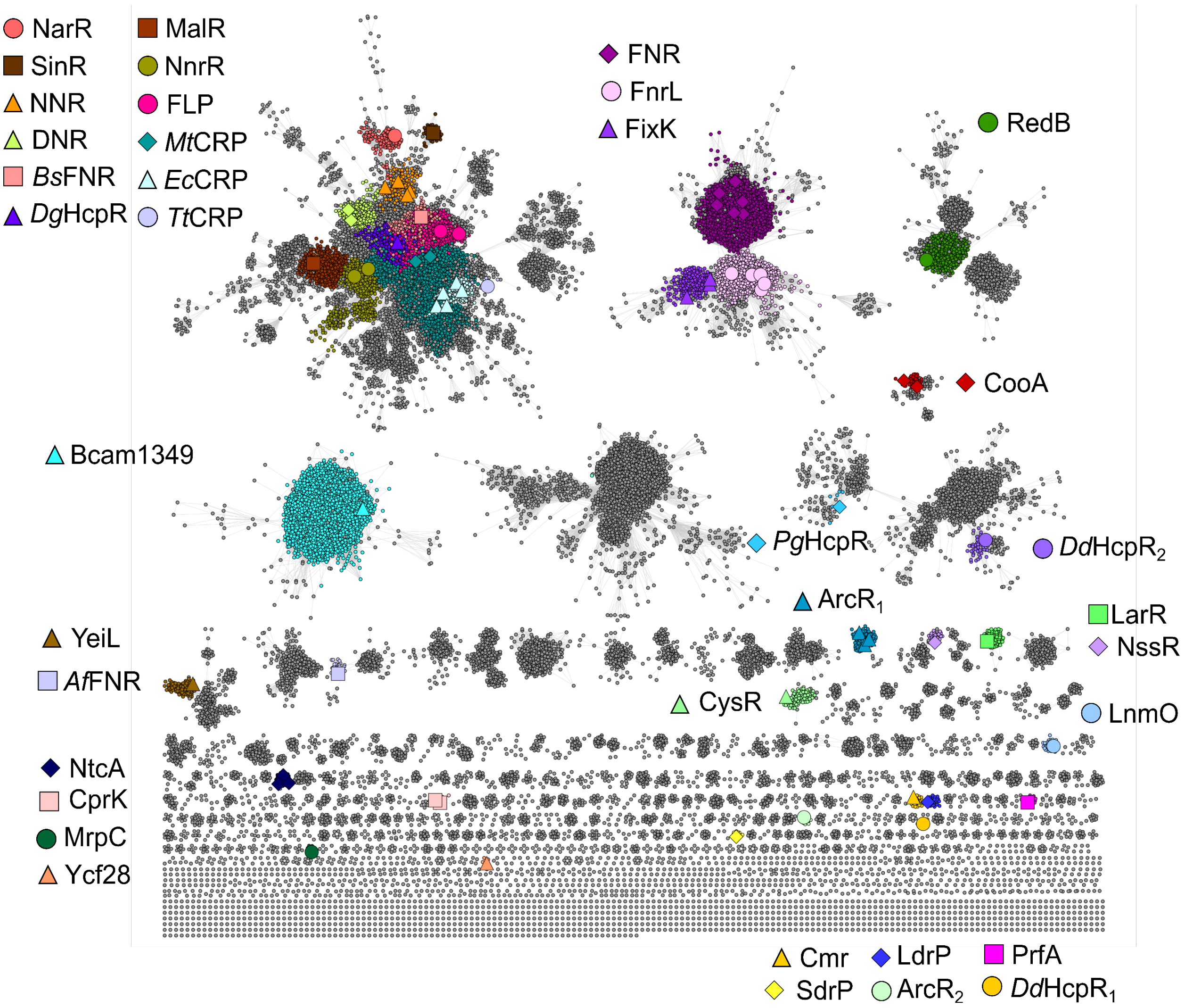

Supplemental Figure S2. A 50% representative sequence similarity network of the CRP/FNR superfamily at an alignment score cutoff of 40. Nodes are colored according to their isofunctional clustering (Figure S1). In comparison to Figure S1, this depiction of the CRP/FNR superfamily highlights relationships between isofunctional clusters. Notably, diverse isofunctional clusters with little regulatory overlap conjoin to generate the largest cluster. Even at this less stringent cutoff score, many clusters are entirely gray, suggesting that varied CRP/FNR proteins that are distantly related to the characterized members of this superfamily have yet to be studied. A list of characterized CRP/FNR proteins that were mapped onto this sequence similarity network is provided in Supplementary Table S1.

Supplemental Table S1. List of the characterized CRP/FNR proteins that are mapped onto the sequence similarity network in Figure 1, Supplemental Figure S1 and Supplemental Figure S2. These proteins have been characterized for some aspect of their functional ability (gene regulation, DNA binding, small molecule sensing, etc.) The reference list is not exhaustive for each protein.

Cluster	Protein	UniProt ID	Reference
*Ec*CRP	CRP	P0ACJ8	(1)
	CLP	Q4UZF6	(3)
	CRP	P29281	(5)
	CRP	O05689	(7)
	vfr	P55222	(8, 9)
*Mt*CRP	GlxR	Q79VI7	(11)
	CRP	P9WMH3	(13)
*Tt*CRP	CRP	Q5SID7	(15)
FNR	ANR	P23926	(17)
	BTR	Q08530	(19)
	FnrP	O31204	(21)
	HlyX	P23619	(23)
	EtrA	P46148	(26)
	FNR	Q70ET4	(28)
*Bs*FNR	BsFNR	P46908	(30)
*Af*FNR	FNR	A0A5P9XRJ7	(32)
FnrL	FixK	P29286	(34)
	AadR	Q01980	(36)
	FnrL	P51007	(38)
	FnrP	Q51677	(40)
	FnrN	P24290	(43)
FixK	FixK$_2$	O69245	(45)
	FixK	P26488	(47)
	FixK	P13295	(49)
NtcA	NtcA	P29283	(51)
	NtcA	P33779	(53)
	NtcA	P0A4U6	(55)
NNR	HbaR	G3XCQ9	(56)
	NNR	Q51661	(58)
	DnrD	A8LM13	(60)
	DnrE	A8LM15	(60)
DNR	DNR	Q51441	(61)

Cluster	Protein	UniProt ID	Reference
ArcR1	ArcR	Q65D84	(2)
	ArcR	Q93K65	(4)
	ArcR	Q8RMP2	(6)
ArcR2	ArcR	Q2FUY1	(10)
FLP	FLP	P29284	(12)
	FLP	A2RJ46	(14)
CooA	CooA	P72322	(16)
	CooA	Q3AB29	(18)
CprK	CprK	Q8RPJ2	(20)
	CprK	B8FW11	(22)
NnrR	NnrR	H7C6M6	(24, 25)
MalR	MalR	G8JZS9	(27)
YeiL	YeiL	P0A9E9	(29)
LarR	LarR	F9USS8	(31)
NarR	NarR	Q93PW2	(33)
SinR	SinR	H0H895	(35)
CysR	CysR	Q55854	(37)
NssR	NssR	Q0PB47	(39)
Cmr	Cmr	P9WMH5	(41, 42)
LdrP	LdrP	Q746J8	(44)
SdrP	SdrP	Q5SIL0	(46)
LnmO	LnmO	Q8GGN7	(48)
MrpC	MrpC	Q1D244	(50)
Ycf28	Ycf28	Q1XDE5	(52)
DdHcpR2	HcpR2	B8J265	(54)
DdHcpR1	HcpR	B8J4I1	(54)
DgHcpR	HcpR	T2GAL9	(57)
PgHcpR	HcpR	Q7MVK4	(59)

	DnrD	Q2KJM7	(62)
	DnrE	Q9X7J7	(62)

References used to generate Supplementary Table S1

[1] Schultz SC, Shields GC, Steitz TA. (1991) Crystal Structure of a CAP-DNA Complex: The DNA is Bent by 90°. *Science 253*, 1001–1007.

[2] Maghnouj A, Abu-Bakr AAW, Baumberg S, Stalon V, Wauven C Van der. (2000) Regulation of anaerobic arginine catabolism in Bacillus licheniformis by a protein of the Crp/Fnr family. *FEMS Microbiol Lett 191*, 227–234.

[3] Tao F, He YW, Wu DH, Swarup S, Zhang LH. (2010) The cyclic nucleotide monophosphate domain of Xanthomonas campestris global regulator Clp defines a new class of cyclic di-GMP effectors. *J Bacteriol 192*, 1020–1029.

[4] Barcelona-Andrés B, Marina A, Rubio V. (2002) Gene structure, organization, expression, and potential regulatory mechanisms of arginine catabolism in Enterococcus faecalis. *J Bacteriol 184*, 6289–6300.

[5] Chandler MS. (1992) The gene encoding cAMP receptor protein is required for competence development in Haemophilus influenzae Rd. *Proc Natl Acad Sci USA 89*, 1626–1630.

[6] Zúñiga M, Miralles M del C, Pérez-Martínez G. (2002) The product of arcR, the sixth gene of the arc operon of Lactobacillus sakei, is essential for expression of the arginine deiminase pathway. *Appl Environ Microbiol 68*, 6051–6058.

[7] Zhao X, Liu Q, Xiao K, Hu Y, Liu X, Li Y, Kong Q. (2016) Identification of the crp gene in avian Pasteurella multocida and evaluation of the effects of crp deletion on its

phenotype, virulence and immunogenicity. *BMC Microbiol 16*, 1–13.

[8] Kanack KJ, Runyen-Janecky LJ, Ferrell EP, Suh SJ, West SEH. (2006) Characterization of DNA-binding specificity and analysis of binding sites of the Pseudomonas aeruginosa global regulator, Vfr, a homologue of the Escherichia coli cAMP receptor protein. *Microbiology 152*, 3485–3496.

[9] West SEH, Sample AK, Runyen-Janecky LJ. (1994) The vfr gene product, required for Pseudomonas aeruginosa exotoxin A and protease production, belongs to the cyclic AMP receptor protein family. *J Bacteriol 176*, 7532–7542.

[10] Makhlin J, Kofman T, Borovok I, Kohler C, Engelmann S, Cohen G, Aharonowitz Y. (2007) Staphylococcus aureus ArcR controls expression of the arginine deiminase operon. *J Bacteriol 189*, 5976–5986.

[11] Townsend PD, Jungwirth B, Pojer F, Bußmann M, Money VA, Cole ST, Pühler A, Tauch A, Bott M, Cann MJ, Pohl E. (2014) The Crystal Structures of Apo and cAMP-Bound GlxR from Corynebacterium glutamicum Reveal Structural and Dynamic Changes upon cAMP Binding in CRP/FNR Family Transcription Factors. *PLoS One 9*, e113265.

[12] Gostick D 0, Green J, Irvine AS, Gasson2 MJ, Guest1 JR. A novel regulatory switch mediated by the FNR-like protein of Lactobacillus casei *Microbiology*.

[13] Bai G, McCue LA, McDonough KA. (2005) Characterization of Mycobacterium tuberculosis Rv3676 (CRPMt), a cyclic AMP receptor protein-like DNA binding protein. *J Bacteriol 187*, 7795–7804.

[14] Scott C, Guest JR, Green J. (2002) Characterization of the Lactococcus lactis transcription factor FlpA and demonstration of an in vitro switch. *Mol Microbiol 35*,

1383–1393.

[15] Shinkai A, Kira S, Nakagawa N, Kashihara A, Kuramitsu S, Yokoyama S. (2007) Transcription activation mediated by a cyclic AMP receptor protein from Thermus thermophilus HB8. *J Bacteriol 189*, 3891–3901.

[16] Shelver D, Kerby RL, He Y, Roberts GP. (1997) CooA, a CO-sensing transcription factor from Rhodospirillum rubrum, is a CO-binding heme protein. *Proc Natl Acad Sci USA1997/10/23. 94*, 11216–11220.

[17] Sawers RG. (1991) Identification and molecular characterization of a transcriptional regulator from Pseudomonas aeruginosa PAO1 exhibiting structural and functional similarity to the FNR protein of Escherichia coli. *Mol Microbiol 5*, 1469–1481.

[18] Youn H, Kerby RL, Conrad M, Roberts GP. (2004) Functionally critical elements of CooA-related CO sensors. *J Bacteriol 186*, 1320–1329.

[19] Bannan JD, Moran MJ, MacInnes JI, Soltes GA, Friedman RL. (1993) Cloning and characterization of btr, a Bordetella pertussis gene encoding an FNR-like transcriptional regulator. *J Bacteriol 175*, 7228–7235.

[20] Kemp LR, Dunstan MS, Fisher K, Warwicker J, Leys D (2013) The transcriptional regulator CprK detects chlorination by combining direct and indirect readout mechanisms. *Philos Trans R Soc B Biol Sci 368*, 20120323.

[21] Uhlich GA, McNamara PJ, Iandolo JJ, Mosier DA. (2000) FnrP interactions with the Pasteurella haemolytica leukotoxin promoter. *FEMS Microbiol Lett 186*, 73–77.

[22] Levy C, Pike K, Heyes DJ, Joyce MG, Gabor K, Smidt H, van der Oost J, Leys D. (2008) Molecular basis of halorespiration control by CprK, a CRP-FNR type transcriptional regulator. *Mol Microbiol2008/08/23. 70*, 151–167.

[23] Green J, Baldwin ML. (1997) HlyX, the FNR homologue of *Actinobacillus pleuropneumoniae* , is a [4Fe–4S]-containing oxygen-responsive transcription regulator that anaerobically activates FNR-dependent Class I promoters via an enhanced AR1 contact. *Mol Microbiol 24*, 593–605.

[24] Tosques IE, Shi J, Shapleigh JP. (1996) Cloning and characterization of nnrR, whose product is required for the expression of proteins involved in nitric oxide metabolism in Rhodobacter sphaeroides 2.4.3. *J Bacteriol 178*, 4958–4964.

[25] Jiménez-Leiva A, Cabrera JJ, Bueno E, Torres MJ, Salazar S, Bedmar EJ, Delgado MJ, Mesa S. (2019) Expanding the Regulon of the Bradyrhizobium diazoefficiens NnrR Transcription Factor: New Insights Into the Denitrification Pathway. *Front Microbiol 10*, 1926.

[26] Saffarini DA, Nealson KH. (1993) Sequence and genetic characterization of etrA, an fnr analog that regulates anaerobic respiration in Shewanella putrefaciens MR-1. *J Bacteriol 175*, 7938–7944.

[27] Cho KH, Cho D, Wang GR, Salyers AA. (2001) New regulatory gene that contributes to control of Bacteroides thetaiotaomicron starch utilization genes. *J Bacteriol 183*, 7198–7205.

[28] Mettert EL, Kiley PJ. (2018) Reassessing the Structure and Function Relationship of the O2 Sensing Transcription Factor FNR. *Antioxidants Redox Signal 29*, 1830–1840.

[29] Anjum MF, Green J, Guest JR. (2000) Yeil, the third member of the CRP-FNR family in Escherichia coli. *Microbiology 146*, 3157–3170.

[30] Esbelin J, Jouanneau Y, Duport C. (2012) Bacillus cereus Fnr binds a [4Fe-4S] cluster and forms a ternary complex with ResD and PlcR. *BMC Microbiol 12*, 125.

[31] Desguin B, Goffin P, Bakouche N, Diman A, Viaene E, Dandoy D, Fontaine L, Hallet B, Hols P. (2015) Enantioselective regulation of lactate racemization by LarR in Lactobacillus plantarum. *J Bacteriol 197*, 219–230.

[32] Osorio H, Mettert E, Kiley P, Dopson M, Jedlicki E, Holmes DS. (2019) Identification and Unusual Properties of the Master Regulator FNR in the Extreme Acidophile Acidithiobacillus ferrooxidans. *Front Microbiol 10*, 1642.

[33] Wood NJ, Alizadeh T, Bennett S, Pearce J, Ferguson SJ, Richardson DJ, Moir JWB. (2001) Maximal expression of membrane-bound nitrate reductase in Paracoccus is induced by nitrate via a third fnr-like regulator named NarR. *J Bacteriol 183*, 3606–3613.

[34] Anthamatten D, Scherb B, Hennecke H. (1992) Characterization of a fixLJ-regulated Bradyrhizobium japonicum gene sharing similarity with the Escherichia coli fnr and Rhizobium meliloti fixK genes. *J Bacteriol 174*, 2111–2120.

[35] Ramey BE, Matthysse AG, Fuqua C. (2004) The FNR-type transcriptional regulator SinR controls maturation of Agrobacterium tumefaciens biofilms. *Mol Microbiol 52*, 1495–1511.

[36] Dispensa M, Thomas CT, Kim MK, Perrotta JA, Gibson J, Harwood CS. (1992) Anaerobic growth of Rhodopseudomonas palustris on 4-hydroxybenzoate is dependent on AadR, a member of the cyclic AMP receptor protein family of transcriptional regulators. *J Bacteriol 174*, 5803–5813.

[37] Rückert C, Milse J, Albersmeier A, Koch DJ, Pühler A, Kalinowski J. (2008) The dual transcriptional regulator CysR in Corynebacterium glutamicum ATCC 13032 controls a subset of genes of the McbR regulon in response to the availability of sulphide

acceptor molecules. *BMC Genomics 9*, 1–18.

[38] Zeilstra-Ryalls JH, Kaplan S. (1995) Aerobic and anaerobic regulation in Rhodobacter sphaeroides 2.4.1: the role of the fnrL gene. *J Bacteriol 177*, 6422–6431.

[39] Elvers KT, Turner SM, Wainwright LM, Marsden G, Hinds J, Cole JA, Poole RK, Penn CW, Park SF. (2005) NssR, a member of the Crp-Fnr superfamily from Campylobacter jejuni, regulates a nitrosative stress-responsive regulon that includes both a single-domain and a truncated haemoglobin. *Mol Microbiol 57*, 735–750.

[40] Hutchings MI, Crack JC, Shearer N, Thompson BJ, Thomson AJ, Spiro S. (2002) Transcription factor FnrP from Paracoccus denitrificans contains an iron-sulfur cluster and is activated by anoxia: Identification of essential cysteine residues. *J Bacteriol 184*, 503–508.

[41] Smith LJ, Bochkareva A, Rolfe MD, Hunt DM, Kahramanoglou C, Braun Y, Rodgers A, Blockley A, Coade S, Lougheed KEA, Hafneh A, Glenn SM, Crack JC, Le Brun NE, Jos´ J, Saldanha JW, Makarov V, Nobeli I, Arnvig K, Mukamolova G V, Buxton RS, Green J. (2017) Cmr is a redox-responsive regulator of DosR that contributes to M. tuberculosis virulence. *Nucleic Acids Res 45*, 6600–6612.

[42] Ranganathan S, Cheung J, Cassidy M, Ginter C, Pata JD, Mcdonough KA. (2017) Novel structural features drive DNA binding properties of Cmr, a CRP family protein in TB complex mycobacteria. *Nucleic Acids Res 46*, 403–420.

[43] Gutiérrez D, Hernando Y, Palacios JM, Imperial J, Ruiz-Argüeso T. (1997) FnrN controls symbiotic nitrogen fixation and hydrogenase activities in Rhizobium leguminosarum biovar viciae UPM791. *J Bacteriol 179*, 5264–5270.

[44] Takano H, Agari Y, Hagiwara K, Watanabe R, Yamazaki R, Beppu T, Shinkai A, Ueda

K. (2014) LdrP, a cAMP receptor protein/FNR family transcriptional regulator, serves as a positive regulator for the light-inducible gene cluster in the megaplasmid of Thermus thermophilus. *Microbiol (United Kingdom) 160*, 2650–2660.

[45] Bonnet M, Kurz M, Mesa S, Briand C, Hennecke H, Grütter MG. (2013) The structure of Bradyrhizobium japonicum transcription factor FixK 2 unveils sites of DNA binding and oxidation. *J Biol Chem 288*, 14238–14246.

[46] Agari Y, Kashihara A, Yokoyama S, Kuramitsu S, Shinkai A. (2008) Global gene expression mediated by Thermus thermophilus SdrP, a CRP/FNR family transcriptional regulator. *Mol Microbiol 70*, 60–75.

[47] Kaminski PA, Mandon K, Arigoni F, Desnoues N, Elmerich C. (1991) Regulation of nitrogen fixation in Azorhizobium caulinodans: identification of a fixK-Vike gene, a positive regulator of nifA. *Mol Microbiol 5*, 1983–1991.

[48] Huang Y, Yang D, Pan G, Tang GL, Shen B. (2016) Characterization of LnmO as a pathway-specific Crp/Fnr-type positive regulator for leinamycin biosynthesis in Streptomyces atroolivaceus and its application for titer improvement. *Appl Microbiol Biotechnol 100*, 10555–10562.

[49] Batut J, Daveran-Mingot ML, David M, Jacobs J, Garnerone AM, Kahn D. (1989) fixK, a gene homologous with fnr and crp from Escherichia coli, regulates nitrogen fixation genes both positively and negatively in Rhizobium meliloti. *EMBO J 8*, 1279–1286.

[50] Feeley BE, Bhardwaj V, Mclaughlin PT, Diggs S, Blaha GM, Higgs PI. (2019) An amino-terminal threonine/serine motif is necessary for activity of the Crp/Fnr homolog, MrpC and for Myxococcus xanthus developmental robustness. *Mol Microbiol 112*, 1531–1551.

[51] Llácer JL, Espinosa J, Castells MA, Contreras A, Forchhammer K, Rubio V. (2010) Structural basis for the regulation of NtcA-dependent transcription by proteins PipX and PII. *Proc Natl Acad Sci USA 107*, 15397–15402.

[52] Kawakami T, Sakaguchi K, Takechi K, Takano H, Takio S. Ammonium Induced Expression of the Red Algal Chloroplast Gene Ycf18, a Putative Homolog of the Cyanobacterial NblA Gene Involved in Nitrogen Deficiency-Induced Phycobilisome Degradation https://doi.org/10.1271/bbb.80662.

[53] García-Domínguez M, Reyes JC, Florencio FJ. (2000) NtcA represses transcription of gifA and gifB, genes that encode inhibitors of glutamine synthetase type I from Synechocystis sp. PCC 6803. *Mol Microbiol 35*, 1192–1201.

[54] Cadby IT, Ibrahim SA, Faulkner M, Lee DJ, Browning D, Busby SJ, Lovering AL, Stapleton MR, Green J, Cole JA. (2016) Regulation, sensory domains and roles of two *Desulfovibrio desulfuricans* ATCC27774 Crp family transcription factors, HcpR1 and HcpR2, in response to nitrosative stress. *Mol Microbiol 102*, 1120–1137.

[55] Zhao MX, Jiang YL, He YX, Chen YF, Teng Y Bin, Chen Y, Zhang CC, Zhou CZ. (2010) Structural basis for the allosteric control of the global transcription factor NtcA by the nitrogen starvation signal 2-oxoglutarate. *Proc Natl Acad Sci USA 107*, 12487–12492.

[56] Egland PG, Harwood CS. (2000) HbaR, a 4-hydroxybenzoate sensor and FNR-CRP superfamily member, regulates anaerobic 4-hydroxybenzoate degradation by Rhodopseudomonas palustris. *J Bacteriol 182*, 100–106.

[57] da Silva SM, Amaral C, Neves SS, Santos C, Pimentel C, Rodrigues-Pousada C. (2015) An HcpR paralog of *Desulfovibrio gigas* provides protection against nitrosative stress.

FEBS Open Bio 5, 594–604.

[58] Van Spanning RJM, De Boer APN, Reijnders WNM, Spiro S, Westerhoff H V., Stouthamer AH, Van der Oost J. (1995) Nitrite and nitric oxide reduction in Paracoccus denitrificans is under the control of NNR, a regulatory protein that belongs to the FNR family of transcriptional activators. *FEBS Lett 360*, 151–154.

[59] Lewis JP, Yanamandra SS, Anaya-Bergman C. (2012) HcpR of Porphyromonas gingivalis is required for growth under nitrosative stress and survival within host cells. *Infect Immun 80*, 3319–3331.

[60] Ebert M, Laaß S, Thürmer A, Roselius L, Eckweiler D, Daniel R, Härtig E, Jahn D. (2017) FnrL and Three Dnr regulators are used for the metabolic adaptation to low oxygen tension in Dinoroseobacter shibae. *Front Microbiol 8*, 642.

[61] Giardina G, Castiglione N, Caruso M, Cutruzzolà F, Rinaldo S. (2011) The Pseudomonas aeruginosa DNR transcription factor: light and shade of nitric oxide-sensing mechanisms. *Biochem Soc Trans 39*, 294–298.

[62] Vollack KU, Härtig E, Körner H, Zumft WG. (1999) Multiple transcription factors of the FNR family in denitrifying Pseudomonas stutzeri: Characterization of four fnr-like genes, regulatory responses and cognate metabolic processes. *Mol Microbiol 31*, 1681–1694.

Supplemental Table S5. Primers used in deletion construction of Δ*redB*

Δ*redB*	ACATGCATGCTCATGGTCGGCGGCTCTGACG	Upstream Forward
Δ*redB*	CTAGTCTAGACCAGTCCATGCGTCTTATCTAATCACCC	Upstream Reverse
Δ*redB*	CTAGTCTAGAGAGGCTTTGTGACGAGGTCACCG	Downstream Forward
Δ*redB*	CGCGGATCCCTGGATGAAGGCAATCCTGCCGC	Downstream Reverse
